# Diversity and associations of parasites, parasitoids, and nest-associated organisms of *Vespa mandarinia* (Hymenoptera: Vespidae) in South Korea

**DOI:** 10.7717/peerj.21240

**Published:** 2026-05-19

**Authors:** Bia Park, Jaehee Kim, Chris Looney, Ohseok Kwon, Moon Bo Choi

**Affiliations:** 1 Department of Biology Education, Kyungpook National University, Daegu, Republic of Korea; 2 Institute for Phylogenomics and Evolution, Kyungpook National University, Daegu, Republic of Korea; 3 Department of Applied Biology, Kyungpook National University, Daegu, Republic of Korea; 4 Washington State Department of Agriculture, Tumwater, Washington, United States; 5 Department of Plant Medicine, Kyungpook National University, Daegu, Republic of Korea; 6 Institute of Agricultural Science and Technology, Kyungpook National University, Daegu, Republic of Korea

**Keywords:** *Vespa mandarinia*, DNA barcoding, Nest-associated organisms, Parasites and parasitoids, Symbiotic interactions

## Abstract

The northern giant hornet (*Vespa mandarinia* Smith, 1852) is a dominant eusocial wasp species in East Asia; however, knowledge of organisms associated with its colonies, including parasites and other nest-associated taxa, remains limited. To address this gap, this study investigated the diversity and ecological roles of organisms associated with *V. mandarinia* colonies and individuals in South Korea using a combination of morphological examination and DNA barcoding analyses. A total of seven associated species were identified: *Xenos moutoni* (du Buysson, 1903) (Strepsiptera: Xenidae); *Volucella suzukii* Matsumura, 1916 and *Vo*. *coreana* Shiraki, 1930 (Diptera: Syrphidae); *Pyralis regalis* (Denis & Schiffermüller, 1775) (Lepidoptera: Pyralidae); *Pheromermis vesparum* Kaiser, 1987 (Nematoda: Mermithidae); *Quedius pectinatus* (Sharp, 1874) (Coleoptera: Staphylinidae); and *Hermetia illucens* (Linnaeus, 1758) (Diptera: Stratiomyidae). These species exhibited distinct ecological associations with *V. mandarinia*. *X*. *moutoni* was confirmed as an obligate endoparasite that induces behavioral and reproductive alterations in its host. *Vo*. *suzukii*, *Q*. *pectinatus*, and *H*. *illucens* were primarily associated with nest-derived detritus beneath hornet nests and exhibited scavenger-like behavior, with no evidence of direct predation on host individuals, suggesting predominantly commensal associations. In contrast, *Vo. coreana* was consistently associated with the comb and nest cells, suggesting a more exploitative ecological strategy. *P*. *regalis* primarily consumed nest material and meconium and occupied an intermediate ecological position between a facultative predator and a nest-associated organism. *Ph*. *vesparum*, recorded for the first time in Korea and Asia, was detected in only a single *V. mandarinia* individual, suggesting a negligible impact on hornet populations under natural conditions. Overall, this study provides baseline taxonomic and ecological data on parasites and nest-associated organisms associated with *V. mandarinia* in Korea and highlights the ecological complexity of hornet nests as microhabitats that support diverse symbiotic assemblages. These findings serve as a foundation for future research on the life histories, host interactions, and potential functional roles of nest-associated organisms in hornet colony dynamics.

## Introduction

The northern giant hornet (*Vespa mandarinia* Smith, 1852) is the largest species of hornet, with a body length ranging from 30 to 45 mm. It is morphologically distinguished from other hornets by its well-developed head and mandibles; it occupies the ecological position of a top predator (Archer, 2012; Kwon & Choi, 2020). The species is distributed across Southeast Asia (*e.g*., Thailand, Vietnam, and Myanmar) and Northeast Asia (*e.g*., eastern China, Japan, and Korea) (Archer, 2012; Smith-Pardo, Carpenter & Kimsey, 2020). Although *V. mandarinia* typically constructs subterranean nests, it has also been observed nesting in other enclosed spaces, such as hollow tree trunks (Matsuura & Sakagami, 1973; Matsuura, 1984; Matsuura & Yamane, 1990; Looney et al., 2023). In recent years, rapid urbanization and habitat modification have been associated with increased reports of *V. mandarinia* in urban green spaces as well as in natural forest habitats (Choi, Kim & Lee, 2012; Kim, Kim & Song, 2020; Kim & Choi, 2021).

Because of its large body size, highly defensive behavior, and potent venom, *V. mandarinia* poses a significant risk to human safety. It is also an important pest in apiculture, as coordinated group attacks can rapidly destroy honeybee colonies (Matsuura & Yamane, 1990; Ono et al., 1995). Because the species frequently nests underground, accidental disturbance of nest entrances during outdoor activities can trigger mass stinging incidents (Matsuura & Yamane, 1990; Choi, Kim & Kwon, 2019). In Korea, group attacks occur particularly frequently in autumn during the cultural practice of *beolcho*, when vegetation around ancestral graves is cleared, and underground nests may be inadvertently disturbed, sometimes resulting in severe injuries or fatalities (Abe, Kawai & Niwa, 1982; Yanagawa et al., 2007; Choi, Kim & Kwon, 2019; Hirano & Tanikawa, 2020).

The detection of *V. mandarinia* in Washington State (USA) and British Columbia (Canada) in 2019–2020 raised concerns regarding its potential ecological and economic impacts outside its native range (Wilson et al., 2020; Alaniz, Carvajal & Vergara, 2021; Nuñez-Penichet et al., 2021). In response, the Washington State Department of Agriculture implemented intensive surveillance and nest removal efforts, reporting the elimination of four nests (Looney et al., 2023) and subsequently announcing a successful first phase of eradication in late 2024 (WSDA News Releases, 18 December 2024).

In contrast, *V. mandarinia* is widely distributed in Korea, where control measures typically rely on emergency response teams or professional pest-control operators who remove nests following sting incidents (Choi, Kim & Kwon, 2019). Beekeepers also commonly use hornet-attractant traps to reduce hornet pressure at apiaries, and nests discovered near apiaries are often eliminated manually (Jung et al., 2007; Choi & Kwon, 2015). Despite its prevalence and importance, ecological research on *V. mandarinia* in Korea remains limited, and much of our current understanding of its life history and behavior is based on studies conducted in Japan (Matsuura & Sakagami, 1973; Matsuura, 1984; Matsuura & Yamane, 1990).

Notably, information on organisms associated with *V. mandarinia* colonies, including parasites, parasitoids, and other nest-associated taxa, is scarce in both Japan and Korea. These associates are of interest not only as natural enemies and exploiters of hornet hosts, but also as components of the hornet-nest microhabitat that may support diverse ecological interactions. In Japan, previous studies have documented *Xenos* spp. (Strepsiptera: Xenidae) associated with *V. mandarinia* individuals (Matsuura & Yamane, 1990; Makino & Yamashita, 1998) and *Quedius* spp. (Coleoptera: Staphylinidae) occurring in or near *V. mandarinia* nests (Watanabe & Arai, 2015). In Korea, *Volucella* spp. (Diptera: Syrphidae) (Kim & Kim, 1972), *Pyralis* spp. (Lepidoptera: Pyralidae) (Shin et al., 2023), and *Xenos* spp. (Strepsiptera: Xenidae) (Makino, Kawashima & Kosaka, 2011) have been reported in association with *V. mandarinia* colonies or individuals. However, the life histories and ecological roles of these organisms within the hornet-nest environment remain poorly understood.

To address this gap in the literature, this study aimed to identify nest-associated organisms of *V. mandarinia* in Korea using integrated morphological and molecular approaches, and to describe their ecological relationships with hornet colonies. *V. mandarinia* nests were collected from multiple locations across Korea, and additional individuals were obtained using wasp-attractant traps deployed nationwide. Examination of nests and adult hornets revealed seven associated species, including obligate parasites and several nest-associated taxa occupying distinct microhabitats within or beneath hornet nests. Because morphological information for many of these taxa is limited in Korea and species identification based solely on morphology can be unreliable, we integrated preliminary morphological identification with DNA barcoding to improve taxonomic resolution and confirm species-level identifications. Additionally, behavioral observations were conducted to infer the ecological roles of each associated species within or around hornet colonies.

## Materials and Methods

### Sample collection

The collection of *V. mandarinia* symbionts was not conducted as a targeted effort solely for this purpose. Instead, specimens were obtained as part of a broader study conducted from 2017 to 2025 investigating the distribution and behavioral characteristics of *V. mandarinia* in South Korea. During this period, specimens were collected using both trapping and nest sampling (Table 1).

**Table 1:** Collection information for the predator, parasites, parasitoids, and inquilines collected from trapped *Vespa mandarinia* individuals and *V. mandarinia* nests in South Korea from 2017 to 2025.

No.	Identified species	Collection location		Date	Collection method	NCBI No.	Voucher no.	No. of collected samples	Developmental stage	Host sex/caste
1	*Xenos moutoni*	Nae-dong, Daegu	35°58′30.10″N 128°39′36.10″E	2019-05-08	Trap	PV246623	X-1	1	Neotenic form	Female/queen
2	*X. moutoni*			2019-06-15	Trap	PV246624	X-2	1	Neotenic form	Female/worker
3	*X. moutoni*			2019-05-15	Trap	PV246625	X-3	3	Neotenic form	Female/Queen
4	*X. moutoni*	Jikdong-ri, Pocheon	37°45′22.1″N 127°09′49.2″E	2017-08-14	Trap	PV246626	X-4	2	Neotenic form	Female/worker
5	*X. moutoni*	Bukseong-dong, Incheon	37°28′15.70″N 126°35′51.94″E	2020-08-30	Trap	PV246627	X-5	1	Neotenic form	Female/worker
6	*X. moutoni*	Jikdong-ri, Pocheon	37°45′22.1″N 127°09′49.2″E	2017-07-31	Trap	PV246628	X-6	1	Neotenic form	Female/worker
7	*X. moutoni*	Gajeong-ri, Gyeongju	35°53′58.44″N 129° 9′8.70″E	2017-08-25	Trap	PV246629	X-7	1	Neotenic form	Female/worker
8	*X. moutoni*	Sinseon-dong, Busan	35° 5′11.45″N 129° 2′58.59″E	2019-09-06	Trap	PV246631	X-9	1	Neotenic form	Female/worker
9	*Pheromermis vesparum*	Naeseo-ri, Gurye	35°14′30.84″N 127°35′19.76″E	2021-07-09	Trap	PV236151	Ph-1	1	Juvenile	Female/queen
10	*Volucella suzukii*	Tae-ri, Andong	36°37′46.43″N 128°45′53.12″E	2020-10-21	Nest 1	PV252796	V-1	30	Larva	
11	*Vo. suzukii*					PV252797	V-2		Larva	
12	*Vo. suzukii*	Oam-ri, Yecheon	36°39′13.47″N 128°32′5.15″E	2019-10-09	Nest 2	PV252798	V-3	30	Larva	
13	*Vo. suzukii*					PV252799	V-4		Larva	
14	*Vo. suzukii*					PV252800	V-5	1	Adult	
15	*Vo. coreana*					PV252801	V-6	16	Larva	
16	*Vo. suzukii*	Hwabong-dong, Ulsan	35°35′54.69″N 129°22′33.12″E	2021-10-20	Nest 3	PV252802	V-7	30	Larva	
17	*Vo. suzukii*					PV252803	V-8		Larva	
18	*Pyralis regalis*					PV242214	Py-5	15	Larva	
19	*P. regalis*			2022-02-09		–	Py-6	38	Adult	
20	*Quedius pectinatus*			2021-10-20		–	S-1	1	Adult	
21	*Unidentified*	Naedong-ri, Gurye	35°15′33.90″N 127°35′56.55″E	2019-08-30	Nest 4	–	V-9	9	Larva	
22	*P. regalis*					PV242210	Py-1	4	Larva	
23	*Vo. coreana*	Igae-ri, Andong	36°36′54.07″N 128°38′29.80″E	2019-10-07	Nest 5	PV252804	V-10	1	Larva	
24	*P. regalis*					PV242212	Py-3	11	Larva	
25	*X. moutoni*					PV246630	X-8	2	Pupa	
26	*Vo. coreana*	Hahoe-ri, Andong	36°33′8.09″N 128°31′50.12″E	2021-08-13	Nest 6	PV252805	V-11	2	Larva	
27	*Vo. coreana*					PV252806	V-12	1	During pupation	
28	*Vo. coreana*					PV252807	V-13	3	Larva	
29	*Vo. coreana*	Naedong-ri, Gurye	35°14′13.76″N 127°35′24.60″E	2019-09-26	Nest 7	PV252808	V-14	1	Larva	
30	*Vo. coreana*					PV252809	V-15	2	Adult	
31	*P. regalis*					PV242211	Py-2	6	Larva	
32	*P. regalis*	Seonggok-dong, Andong	36°34′9.74″N 128°46′15.56″E	2019-09-20	Nest 8	PV242213	Py-4	4	Larva	
33	*P. regalis*	Gaguri, Andong	36°36′47.89″N 128°46′42.17″E	2021-10-20	Nest 9	PV242215	Py-7	24	Larva	
34	*P. regalis*			2022-01-21		–	Py-8	45	Adult	
35	*Q. pectinatus*	Gudam-ri, Andong	36°32′54.09″N 128°28′10.75″E	2020-10-12	Nest 10	PV241484	S-2	6	Larva	
36	*Q. pectinatus*	Cheolpa-ri, Uiseong	36°21′54.69″N 128°39′34.21″E	2022-09-02	(Rearing) Nest 11	PV241485	S-3	76	Larva	
37	*Q. pectinatus*					PV241486	S-4	11	Adult	
38	*Q. pectinatus*		36°21′54.15″N 128°39′32.82″E	2022-09-20	(Rearing) Nest 12	PV241487	S-5	79	Larva	
39	*Q. pectinatus*	Pungri-ri, Uiseong	36°17′52.12″N 128°38′57.31″E	2022-09-16	Nest 13	–	S-6	2	Adult	
40	*Hermetia illucens*	Arail-dong, Jeju	33°26′47.83″N 126°33′23.50″E	2025-09-30	Trap	PX626602	HeJJA-1	1	Adult	
41	*H. illucens*	Sinwol-ri, Uiseong	36°22′1.14″N 128°38′52.50″E	2025-10-02	(Rearing) Nest 14	PX626604	HeUSA-1	1	Adult	
42	*H. illucens*					PX626605	HeUSA-2	1	Adult	
43	*H. illucens*					PX626606	HeUSL-1	1	Larva	

#### Hornet trapping

Hornet trapping was conducted annually from 2017 to 2021 across multiple regions in South Korea, including Gyeongsangbuk-do, Gyeonggi-do, Busan, Daegu, Incheon, and Jeollabuk-do. In each region, seven traps were deployed every year, totaling 42 traps per year and 210 traps over the entire study period. The hornet traps consisted of a two-chamber system. The primary chamber was a plastic bottle (10 cm width × 10 cm depth × 25 cm height) connected to a secondary chamber, a smaller plastic bottle (5 cm × 5 cm × 15 cm), *via* a 3 cm diameter plastic tube. A 3 cm diameter opening was created in the primary chamber, which contained 250 mL of an attractant solution (70% plum extract + 30% water) along with a fine mesh barrier to prevent hornets from falling into the attractant after entry. The secondary chamber contained 98% ethanol to preserve the specimens. Hornets entering the primary chamber were guided through the plastic tube into the secondary chamber, where they were killed and preserved in ethanol. Traps were deployed from June to October and retrieved monthly. At each collection event, hornets showing evidence of strepsipteran or mermithid parasites were identified and retained for further analysis (Table 1). All host and parasite specimens were preserved in 98% ethanol and stored at −25 °C in a freezer. A total of 5,078 social wasp specimens were collected using traps, of which 437 were *V. mandarinia*. All *V. mandarinia* specimens collected in the traps were examined for evidence of internal parasites.

### Nest collection and colony rearing

Nest collection was conducted between 2019 and 2025 across multiple locations in South Korea, including Andong, Yecheon, Ulsan, Gurye, and Uiseong. All nest-associated organisms found within collected nests were retained for identification and analysis. A total of 14 nests were examined, of which 11 were naturally occurring nests and 3 were rearing nests maintained for behavioral experiments (Table 1).

For the naturally occurring nests, we acknowledge that the presence of nest associates may be influenced by nest condition and colony size (*e.g*., number of combs, number of cells, and adult demographics). However, detailed nest parameters were not systematically recorded during the original sampling period. As described in the Methods section, the collection of *V. mandarinia* nest-associated organisms was conducted as part of a broader ecological study investigating the distribution and behavioral characteristics of the species in South Korea, rather than as a targeted investigation of nest associates. During field sampling, our primary objective was to detect and collect nest-associated organisms, not to quantify colony structural or demographic characteristics.

Furthermore, several naturally occurring nests were collected by experienced wasp collectors for independent purposes or for analyses unrelated to the present study; thus, detailed nest characteristic data were not obtained at the time of collection. Some nests collected in autumn were maintained until winter (*e.g*., to observe the emergence of *Pyralis* adults), but were subsequently destroyed for chemical extraction experiments and other research purposes. The remaining nests were also used in various subsequent experiments, leaving few intact specimens currently available. Consequently, retrospective analysis of detailed nest architectural and colony parameters for the naturally occurring nests was not feasible.

For the rearing nests, field colonies were collected on August 5, 2022 (three combs, 86 workers, and one queen), August 9, 2022 (three combs, 72 workers, and one queen), and August 13, 2025 (three combs, 111 workers, and one queen). During collection, all individuals were captured alive and nest combs were carefully preserved to prevent structural damage. Each collected nest was transferred into a wooden rearing box (47 cm W × 37 cm D × 25 cm H), with the nest comb affixed to the interior ceiling. The box was then sealed, and a single entrance hole (5 cm × 5 cm) was created at the base of one lateral wall. All collected individuals were introduced into the box through this entrance.

The rearing boxes were installed on the ground at outdoor study sites (located in forested areas of Cheolpa-ri and Sinwol-ri, Uiseong-gun). Colonies were provided with honey solution as a carbohydrate source and grasshoppers as a protein source. Following installation, colonies were allowed to settle for approximately 1 day before the entrance was opened the following morning to permit foraging activity. Following an acclimation period of 1 to 2 days, normal foraging behavior was confirmed prior to the commencement of observations.

### Collection and identification of nest-associated organisms

In the following sections, organisms are arranged and described according to systematic (taxonomic) order.

### Genus *Quedius* (Coleoptera: Staphylinidae)

*Quedius* specimens were frequently found on the ground beneath *V. mandarinia* nests during collection, and both adults and larvae were observed. When staphylinid beetles were detected within a nest, they were immediately collected (Table 1).

Adult specimens were identified using the taxonomic key provided by Zhao & Zhou (2015), and their identifications were verified by the staphylinid specialist Dr. S. K. Lee. Larvae could not be identified morphologically owing to a lack of taxonomic information and expertise. Therefore, DNA barcoding was performed for larval identification.

As no genetic reference sequences for this species were available in sequence databases such as NCBI, direct comparison for species confirmation was not feasible. Therefore, larval sequences were compared with those from morphologically identified adults. Genetic distances among these individuals were calculated to confirm species identity.

### Genus *Xenos* (Strepsipteran: Xenidae)

For each parasitized host, the caste and collection details were recorded, and body size was measured. The number and sex of strepsipteran parasites within each host were also documented (Table 1). Morphological identification of strepsipteran parasites was performed using the taxonomic key developed by Nakase & Kato (2013). However, owing to the subtle morphological differences in mandible spines, species identification based solely on morphology was challenging. Therefore, sequencing of the mitochondrial COI “barcode” region was used to supplement species identifications. In cases of multiple parasitism, only one parasite per host was selected for DNA confirmation.

### Genus *Hermetia* (Diptera: Stratiomyidae)

Individuals of the genus *Hermetia* were collected in large numbers from the bottom of a single wooden rearing box (refer to the subsection Nest Collection and Colony Rearing) used for outdoor maintenance of an experimental colony during behavioral experiments on *V. mandarinia* in Uiseong, South Korea, between August and October 2025 (Table 1). In late August, approximately ten first-instar larvae newly hatched from eggs were first observed on the floor of the rearing boxes. Subsequently, larval abundance increased rapidly, reaching several hundred individuals between late September and early October. On 2 October, 53 larvae were collected from these aggregations and preserved in 99% ethanol. The remaining individuals were transported to the rearing facility at Kyungpook National University and maintained in a plastic rearing container (32 cm × 18 cm × 15 cm; length × width × height) for approximately 1 month. After this period, the container was opened, and a total of 69 adult black soldier flies, both alive and dead, were collected.

Larvae and adults were preliminarily identified based on external morphology following Jeong et al. (2018). For final species confirmation, one larva (HeUSL-1), two adults (HeUSA-1 and HeUSA-2), and an additional adult reference specimen of *Hermetia illucens* (Linnaeus, 1758) from Jeju Island, preserved in the Kyungpook National University laboratory collection (HeJJA-1), were subjected to DNA barcoding. Species identity was confirmed through comparative sequence analysis.

### Genus *Volucella* (Diptera: Syrphidae)

Adult, larval, and pupal stages of *Volucella* were collected from *V. mandarinia* nests during nest sampling. Adult individuals were collected when found inside the hornet nests; although they eventually leave, they often remain within the nest for an undetermined period after emerging from the pupal stage. Larvae were observed moving within the comb and on the ground beneath the nests.

After all adult *V. mandarinia* were removed, the remaining *Volucella* larvae and pupae were manually extracted from each nest cell. Larvae present in the soil beneath the nests were collected separately following nest removal.

Notably, the number of *Volucella* larvae found beneath the nests increased sharply between September and October, with approximately 200–500 individuals observed on the ground beneath the nest during this period. As analyzing all collected individuals was not feasible, 30 larvae were selected from these aggregations, of which one to two individuals representing different developmental stages were retained for species identification (V-1 to V-4 and V-7 to V-9) (Table 1). Adult specimens were identified using the taxonomic keys outlined by Choi (2004) and Kim & Kim (1972), and final species identifications were verified by Dr. D. S. Choi. However, taxonomic data on *Volucella* pupae and larvae in Korea is scarce. Therefore, after species identities of adults were confirmed morphologically, the COI region was sequenced to establish a DNA barcode library. Pupae and larvae were preliminarily sorted based on Ohara (1985), and selected individuals were subjected to DNA barcoding. Species identity was confirmed by matching their sequences to those of the morphologically identified adults.

### Genus *Pyralis* (Lepidoptera: Pyralidae)

Pyralid moths were opportunistically collected when larvae or adults were encountered within *V. mandarinia* nests, either during nest collection or while nests were being stored. Because several pyralid moth species exhibit highly similar external larval morphology, one larva per nest was selected for DNA barcoding to confirm species identity. Additionally, two adult specimens were collected during January and February 2022 after larvae maintained in stored nests in the laboratory developed into moths (Table 1). The adult specimens were morphologically identified based on the classification criteria provided by Shin et al. (2023).

### Genus *Pheromermis* (Mermithida: Mermithidae)

A single nematode was observed protruding from the abdomen of a *V. mandarinia* specimen collected from a hornet trap deployed in the Jirisan region of Gurye between July 9 and August 6, 2021 (Table 1). The parasitized hornet specimen was dissected to extract the nematode, which was photographed and subjected to taxonomic identification. Owing to the difficulty in identifying nematodes based on morphology alone, species identification was confirmed using DNA barcoding.

### Specimen photography and documentation

Photographs of symbionts in their natural habitats were captured in the field using a digital camera (D-810; Nikon, Tokyo, Japan). Specimen images for detailed morphological documentation were captured under a Leica M250C stereomicroscope (Leica Microsystems, Wetzlar, Germany).

### DNA extraction, polymerase chain reaction, and sequencing

For molecular identification, genomic DNA was extracted from whole-body tissue samples using the DNeasy Blood & Tissue Kit (Qiagen Co., Hilden, Germany) according to the manufacturer’s protocol. The extracted DNA was stored at −80 °C and subsequently deposited at Kyungpook National University (KNU). Voucher numbers were assigned to each genus as follows: X-1 to X-9 for *Xenos*; V-1 to V-15 for *Volucella*; HeJJA-1, HeUSA-1, 2, and HeUSL-1 for *Hermetia*; Py-1 to Py-5 and Py-7 for *Pyralis*; Ph-1 for *Pheromermis*; and S-2 to S-5 for *Quedius* (Table 1).

A partial fragment of the mitochondrial COI gene was amplified for insect specimens, and the nuclear 18S rRNA gene was amplified for the nematode, using previously reported primer sets (Table 2). The primers LCO1490 and HCO2198 were used for the COI gene (Folmer et al., 1994), and 18S-1F and 18S-5R were used for the 18S rRNA gene (Giribet et al., 1996).

**Table 2:** Information on the primer pairs used in this study, including their sequences, optimal annealing temperatures, and original references.

Gene	Primers		Annealing temperature	Reference
COI	LCO1490	GGTCAACAAATCATAAAGATATTGG	50 °C or 52 °C	Folmer et al. (1994)
	HCO2198	TAAACTTCAGGGTGACCAAAAAATCA		
18S rRNA	18S-1F	TACCTGGTTGATCCTGCCAGTAG	51 °C	Giribet et al. (1996)
	18S-5R	CTTGCAAAGCTGCTTTCGC		

Polymerase chain reaction (PCR) was performed in a total reaction volume of approximately 50 μL. The reaction mixture contained 1 μL of genomic DNA, 5 μL of 10× PCR buffer, 1 μL of 2.5 mM dNTP mixture, 1 μL of each primer (20 μM), 0.25 μL of Taq DNA polymerase (5 U/μL), and 40.8 μL of double-distilled water. The thermocycling conditions consisted of an initial denaturation at 94 °C for 2 min, followed by 35 cycles of denaturation at 94 °C for 30 s, annealing at 50 °C or 52 °C (COI) or 51 °C (18S rRNA) for 30 s, and extension at 72 °C for 1 min. A final extension step was performed at 72 °C for 5 min.

The amplified PCR products were purified using the QIAquick PCR Purification Kit (Qiagen Co., Hilden, Germany) and sequenced by SolGent Co., Ltd. (Daejeon, Republic of Korea), a commercial sequencing service provider, using an ABI Prism 3730 DNA sequencer (PerkinElmer Inc., Rodgau, USA) with the Big Dye Terminator Sequencing Kit (PerkinElmer Inc., Rodgau, USA).

All novel sequences obtained in this study were deposited in the NCBI GenBank database under the assigned accession numbers. COI sequences were registered under PV246623–PV246631 for genus *Xenos* (X-1 to X-9); PV252796–PV252803 and PV252804–PV252809 for genus *Volucella* (V-1 to V-8 and V-10 to V-15, respectively); PX626602 and PX626604–PX626606 for genus *Hermetia* (HeJJA-1, HeUSA-1, 2, HeUSL-1, respectively); PV242210–PV242214 and PV242215 for genus *Pyralis* (Py-1 to Py-5 and Py-7, respectively); and PV241484–PV241487 for genus *Quedius* (S-2 to S-5). The 18S rRNA sequence for genus *Pheromermis* (Ph-1) was deposited under accession number PV236151.

### Phylogenetic analysis

Phylogenetic relationships were inferred using the *COI* and *18S rRNA* gene sequences generated in this study. The nucleotide sequences utilized for phylogenetic reconstruction are listed in Table 3, comprising 38 newly obtained sequences from this study, along with 73 sequences retrieved from the NCBI GenBank database. To generate nucleotide sequence alignments for the five taxa (*Xenos*, *Volucella*, *Hermetia*, *Pyralis*, and *Pheromermis*), taxon-specific sequences were aligned in MEGA 12 (Kumar et al., 2024) using default settings. The alignments were manually inspected to remove ambiguously aligned regions. For rove beetles, for which closely related reference sequences were not available in public databases, genetic distances between the mitochondrial *COI* gene sequences of the newly collected specimens were estimated. Phylogenetic trees were reconstructed using the neighbor-joining (NJ) method in MEGA12 (Kumar et al., 2024). Genetic distances were estimated using the Tamura–Nei (TN93) model with a gamma distribution (+G, shape parameter = 4.00). Nodal support was assessed using 1,000 bootstrap replications.

**Table 3:** Species and sequences (accession nos) used in the COI-based (Insecta) and 18S rRNA-based (Nematoda) phylogenetic analyses.

Taxon		Scientific name	NCBI accession nos.	Note
Insecta	Lepidoptera (Pyralidae)	*Pyralis caustica*	KF395549, KF402803	
		*P. farinalis*	GU088473, HQ546654, JF852990, KX040068, KX043023	
		*P. lienigialis*	HM874785, HM874786	
		*P. manihotalis*	HQ546656, JN277308	
		*P. regalis*	PV242210–PV242215	From this study: voucher nos. Py-1–5, Py-7
		*P. regalis*	MN609086	
		*Pyralis sp*.	KF399665, KF401747, KX862414	
		*Aglossa pinguinalis*	HM874676	Outgroup
	Strepsiptera (Xenidae, Stylopidae)	*Xenos hamiltoni*	JN082807	
		*X. moutoni*	PV246623–PV246631	From this study: voucher nos. X-1–9
		*X. moutoni*	AB759581, LC764846, ON548474	
		*X. oxyodontes*	AB759568, AB759569	
		*X. pecki*	JN082808	
		*X. vesparum*	JN082806	
		*X. vespularum*	AB759583	
		*Stylops advarians*	MT997187	Outgroup
	Diptera (Syrphidae)	*Volucella bombylans*	HM860407, HM860565	
		*Vo. coreana*	PV252801, PV252804–PV252809	From this study: voucher nos. V-6, V-10–15
		*Vo. inanis*	MN622110, MZ610558	
		*Vo. pellucens*	MG823449, MZ628285	
		*Vo. suzukii*	PV252796–PV252800 PV252802–PV252803	From this study: voucher nos. V-1–5, V-7, V-8
		*Vo. trifasciata*	MW473997	
		*Vo. zonaria*	MN622112, MN868847	
		*Graptomyza flavicolis*	JN269907	Outgroup
	Diptera (Stratiomyidae)	*Hermetia flavimaculata*	MW335652	
		*H. illucens*	PX626602, PX626604, PX626605, PX626606	From this study: voucher nos. HeJJA-1, HeUSA-1, 2, HeUSL-1
		*H. illucens*	MT178470, MT178489, MT178507, MT433999	
		*H. inflata*	MT434000	
		*H. transmaculata*	MW335653	
		*Odontomyia pubescens*	MN961477	Outgroup
	Coleoptera (Staphylinidae)	*Quedius pectinatus*	PV241484–PV241487	From this study: voucher nos. S-2–5
Nematoda	Mermithida (Mermithidae)	*Isomermis lairdi*	FN400892–FN400900	
		*Limnomermis sp*.	KJ636371	
		*Mermis nigrescens*	AF036641, KF583882, KF583883	
		*Mermis sp*.	FJ973464	
		Mermithidae sp.	AB647218, AB647220–AB647224, AY284743, FJ040480, KJ636328	
		*Pheromermis vesparum*	PV236151	From this study: voucher no. Ph-1
		*Ph. vesparum*	KR029620, KR029621	
	Araeolaimida (Aulolaimidae)	*Aulolaimus oxycephalus*	KJ636344	Outgroup
	Isolaimida (Isolaimiidae)	*Isolaimium multistriatum*	KJ636343, KJ636356	Outgroup
		*Isolaimium sp*.	AY552971	Outgroup

## Results

### Genus *Quedius* (Coleoptera: Staphylinidae)

From 2020 to 2022, *Quedius* specimens were identified in five nests (Nests 3 and 10–13) collected from Andong, Ulsan, and Uiseong (Table 1). Among these, Nests 3, 10, and 13 were naturally occurring nests, where adults and larvae were collected from the nest floor. In contrast, Nests 11 and 12 were artificial nests maintained in Cheolpari Forest, Uiseong, Gyeongsangbuk-do, for behavioral experiments on *V. mandarinia*. Approximately 70–80 larvae were collected from the floors of these two experimental nests, and approximately 10 adult beetles were collected from Nest 11 (Table 1).

**Figure 1:**
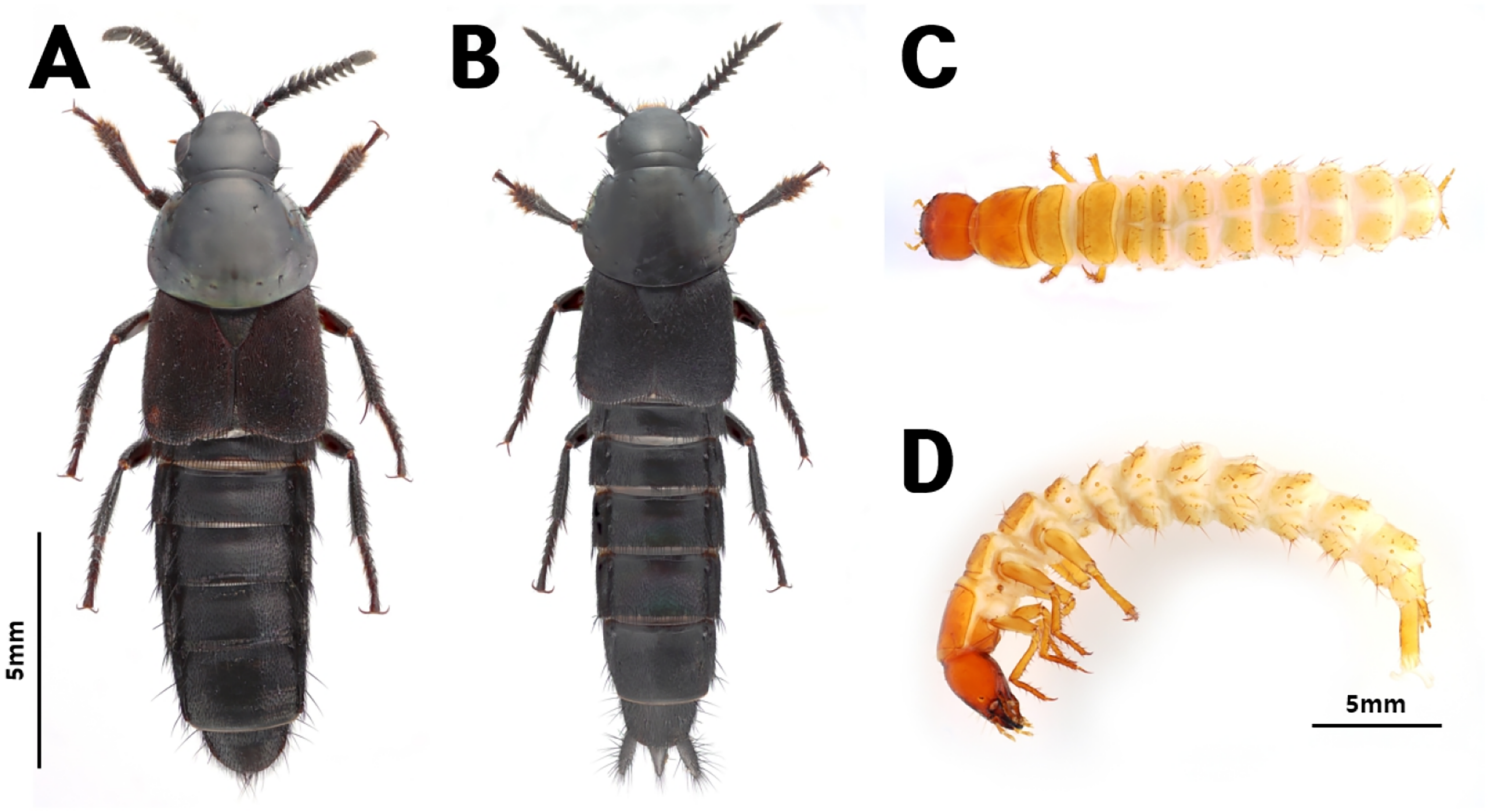
*Quedius pectinatus*. (A) Female and (B) male of adult, and (C) dorsal and (D) lateral views of a larva.

*Quedius* spp. inhabiting the area beneath *V. mandarinia* nests were morphologically identified as *Quedius pectinatus* (Sharp, 1874) (Figs. 1A, 1B). To confirm the identity of larvae (S-2, S-3, S-5) (Figs. 1C, 1D), the COI barcoding region was sequenced from an adult specimen (S-4) and compared with that from the three larval specimens. Genetic distances ranged from 0.00% to 0.49%, confirming that all four specimens belonged to the same species (Table 4).

**Table 4:** Genetic distances among mitochondrial COI sequences from *Quedius* samples.

	S-2 (PV241484)	S-3 (PV241485)	S-4 (PV241486)	S-5 (PV241487)
S-2 (PV241484)				
S-3 (PV241485)	0.0048776858			
S-4 (PV241486)	0.0048776858	0.0000000000		
S-5 (PV241487)	0.0048776858	0.0000000000	0.0000000000	

**Figure 2:**
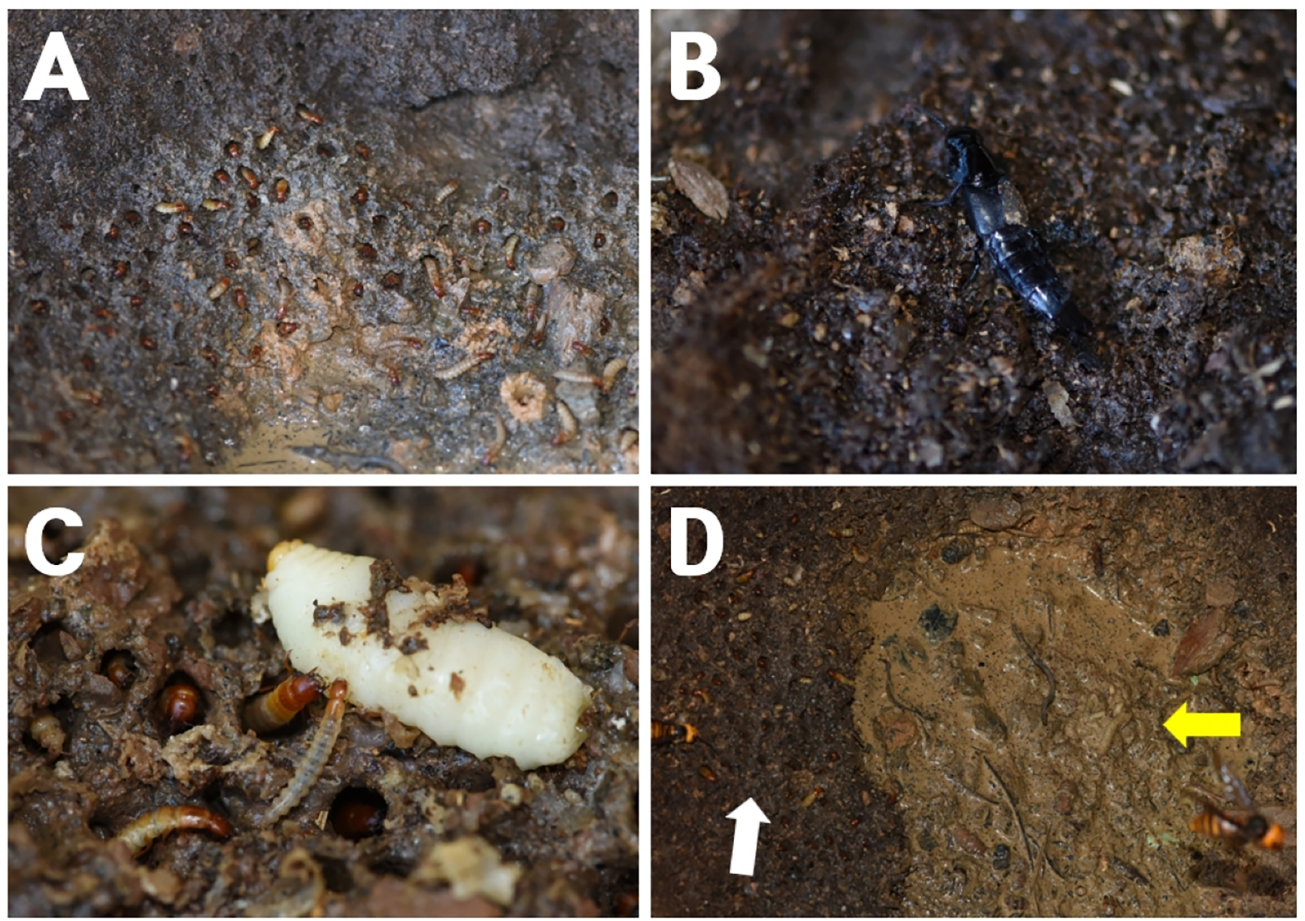
*Quedius pectinatus* inhabiting the ground at the bottom of a *Vespa mandarinia* nest. (A) Numerous *Q. pectinatus* larvae burrow individually into the soil beneath the nest. (B) An adult *Q. pectinatus* actively moves beneath the nest. (C) *Q*. *pectinatus* larvae consume a *V. mandarinia* larva that has been discarded beneath the nest by adult hornets. (D) The central area beneath the nest is occupied by *Vo. suzukii* larvae (yellow arrow), while *Q. pectinatus* larvae reside along the periphery (white arrow), functioning as detritus cleaners in the *V. mandarinia* nest.

Larvae were observed constructing shallow chambers in the detritus beneath the nest floor, embedding the posterior part of the body while keeping the head exposed (Fig. 2A). When dead hornet larvae or adults fell beneath the nest, the larvae emerged and fed on the remains (Fig. 2C). The larvae were mainly distributed along the peripheral areas beneath the nest (Fig. 2D, white arrow), whereas *Vo. suzukii* larvae occupied the central detritus-rich zone (Fig. 2D, yellow arrow).

### Genus *Xenos* (Strepsiptera: Xenidae)

**Figure 3:**
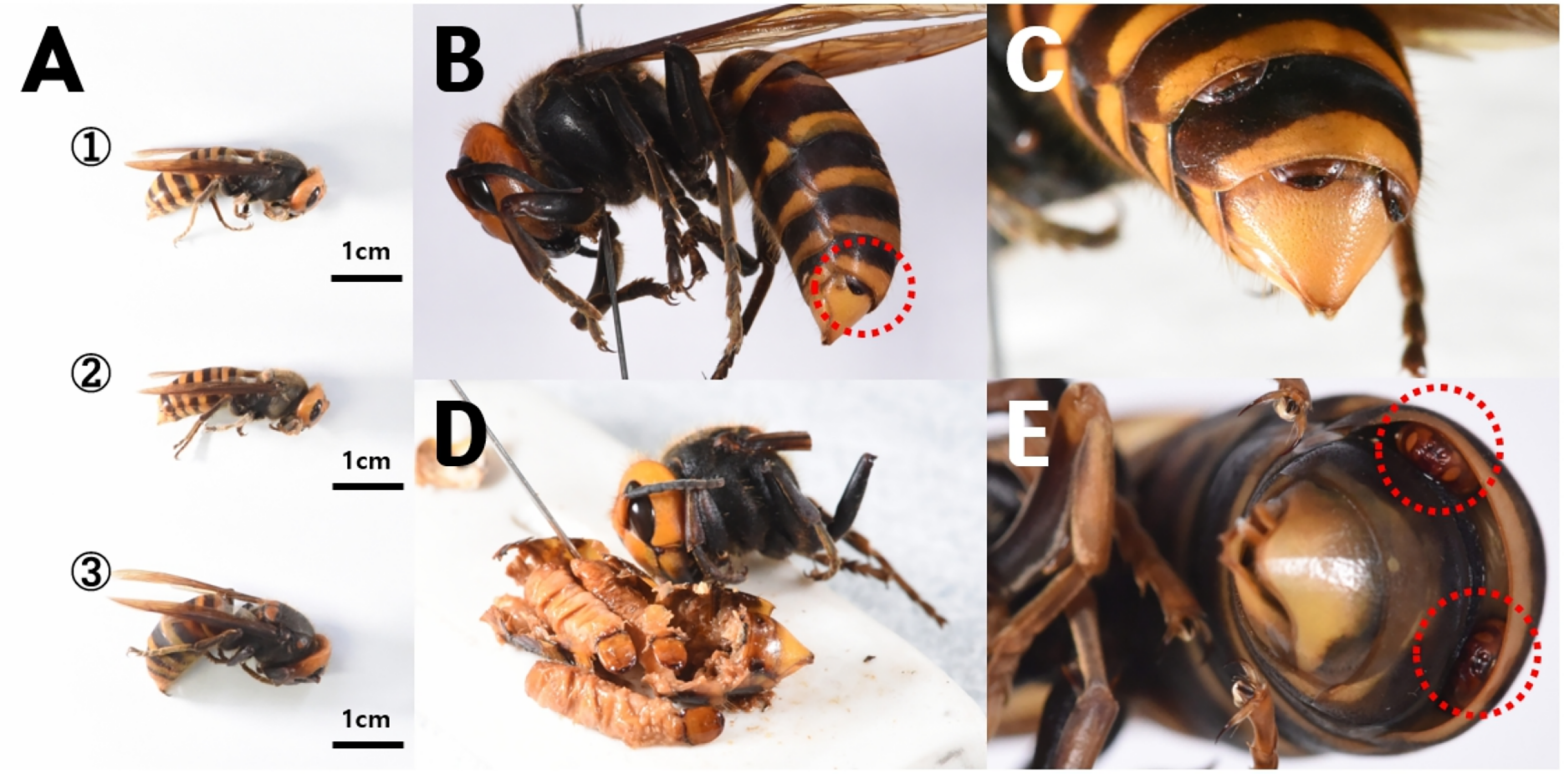
Strepsipteran parasites (*Xenos* sp.) found in the abdomens of adult *V espa mandarinia*. (A) Stylopized workers (①: voucher X-1, size = 3.1 cm; ②: X-2, 2.8 cm) and a queen (③: X-3, 4.0 cm), (B) a female strepsipteran parasite (X-9) parasitizing a wasp, (C) three strepsipterans parasitizing a single *V. mandarinia* queen (X-3), (D) the three parasites shown in (C) (X-3) after extraction from the host, and (E) two male strepsipteran parasites (X-8) parasitizing a single host.

A total of nine stylopized *V. mandarinia* females were collected from traps and nests (Table 1), including two queens and seven workers. From these hosts, 13 strepsipteran parasites were extracted, including 11 females (X-1 to X-7 and X-9) (Fig. 3B) and two males (X-8) (Fig. 3E).

**Figure 4:**
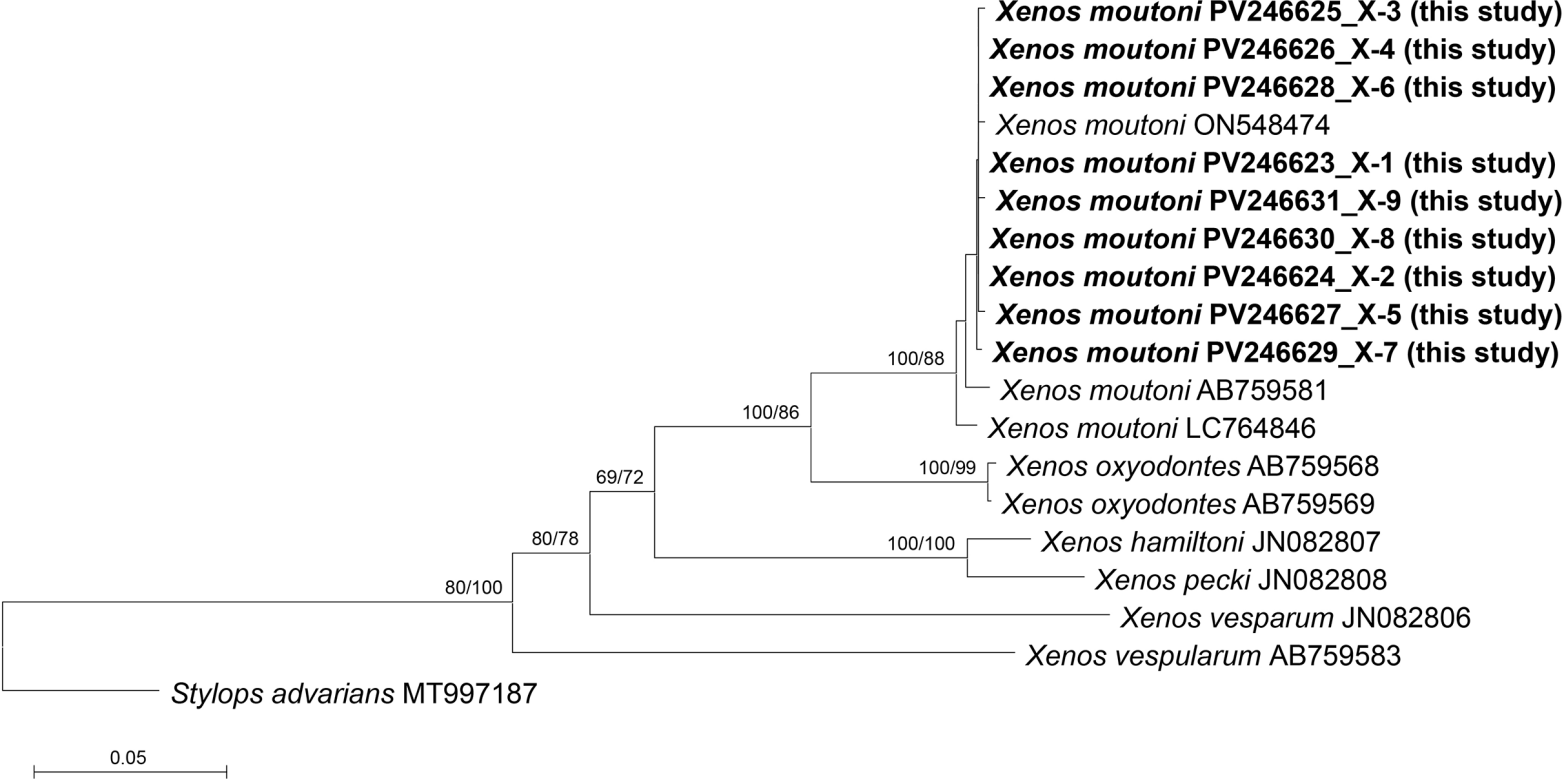
Phylogenetic tree constructed using the neighbor-joining (NJ) method showing the relationships among the 18 aligned *COI* sequences of *Xenos* species. *Stylops advarians* was selected as an outgroup. The numbers above the nodes represent NJ bootstrap values, expressed as percentages (%). Species names are indicated with the sequences’ accession numbers in the NCBI GenBank database, and the newly sequenced individual (in bold) is also labeled with its voucher number (see Table 3 for detailed information).

Notably, the individual from which X-3 was collected, a queen, exhibited multiple parasitism, harboring three female strepsipteran parasites (Figs. 3C, 3D). Similarly, X-4 harbored two female strepsipteran parasites, and X-8 was parasitized by two males (Fig. 3E). DNA barcoding using a single parasite from each host confirmed that all nine parasitized individuals belonged to *X. moutoni* (du Buysson, 1903) (Fig. 4).

**Figure 5:**
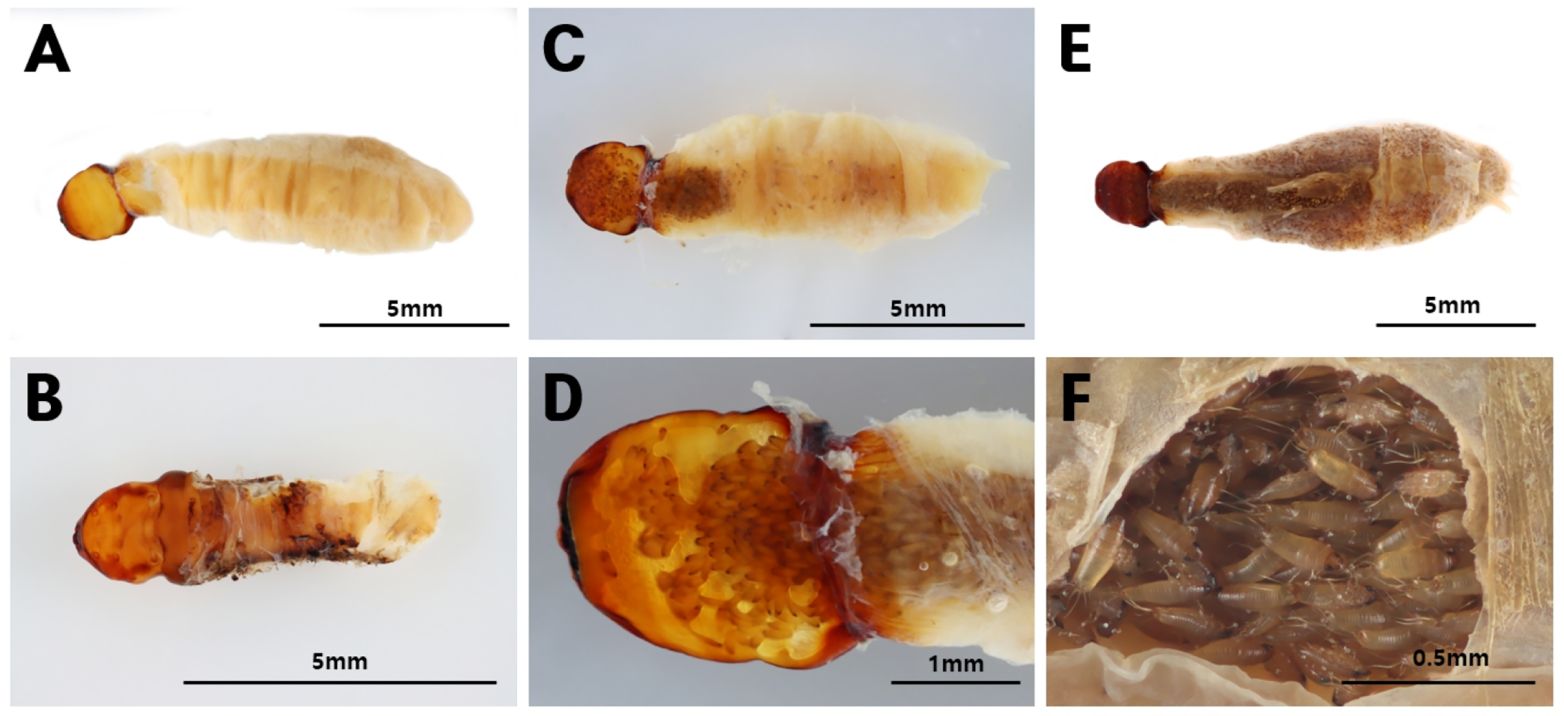
*Xenos moutoni* extracted from *Vespa mandarinia* wasp abdomens. (A) A neotenic female before the development of planidium larvae, (B) A male pupa, (C) planidium larvae developing in the anterior abdomen of a neotenic female, (D) planidium larvae being released through the brood canal opening located on the cephalothorax of a female, (E) a neotenic female with its entire abdomen filled with planidium larvae, and (F) planidium larvae inside a mature female.

Pupal-stage males of *X. moutoni* were observed in several specimens (Fig. 5B). Neotenic females contained developing planidium larvae within the abdomen (Fig. 5F). Larvae were not observed in younger female parasites (Fig. 5A), whereas larvae were present in the anterior abdomen of older females (Fig. 5C) and filled the abdominal cavity in mature females (Fig. 5E). In several cases, larvae were observed emerging through the brood canal (Fig. 5D).

### Genus *Hermetia* (Diptera: Stratiomyidae)

**Figure 6:**
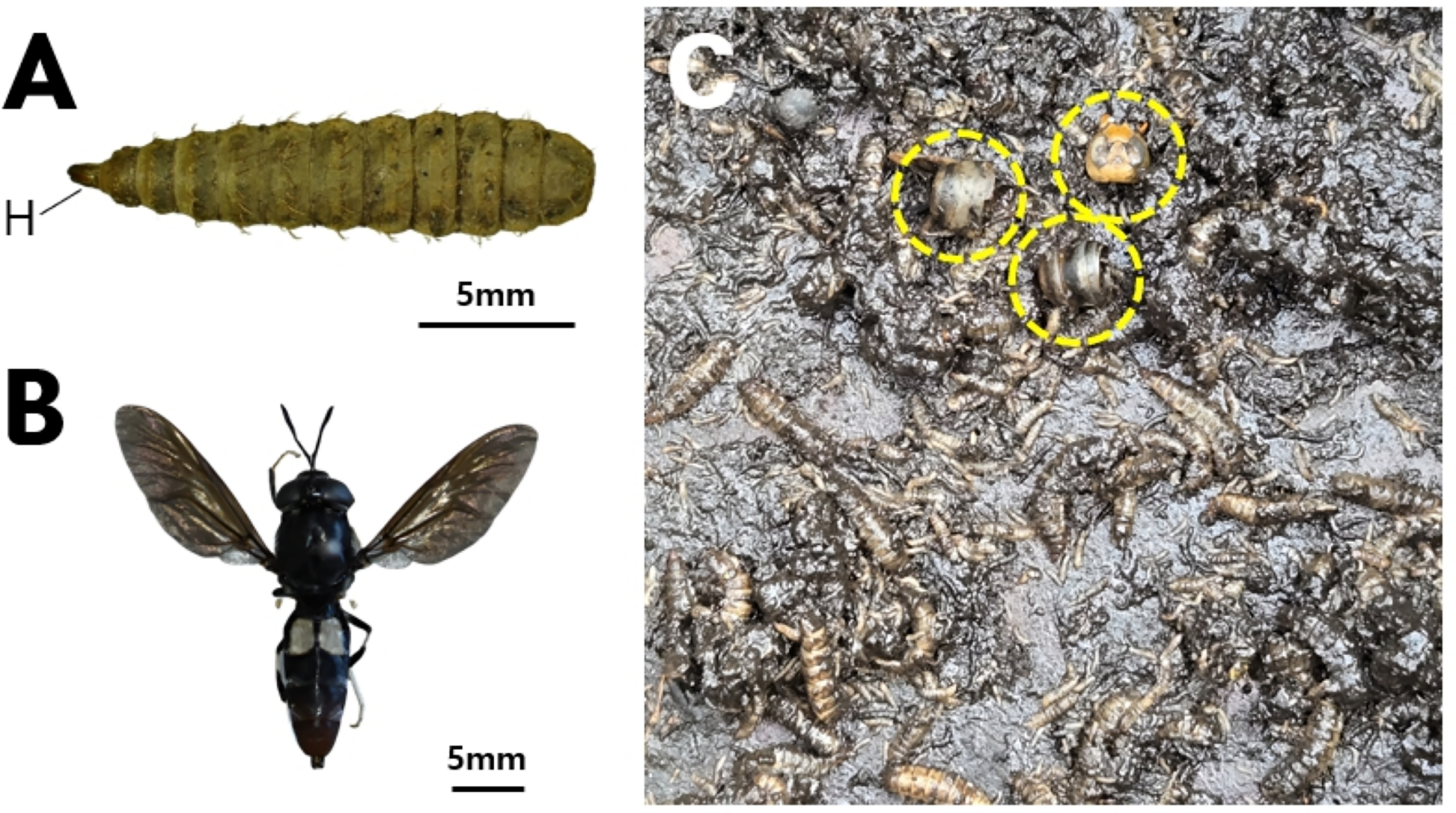
*Hermetia illucens*. (A) Larva (H: head), (B) Adult, and (C) *H. illucens* larvae aggregated on the ground beneath a nest (yellow circle: hornet remains processed by larvae).

**Figure 7:**
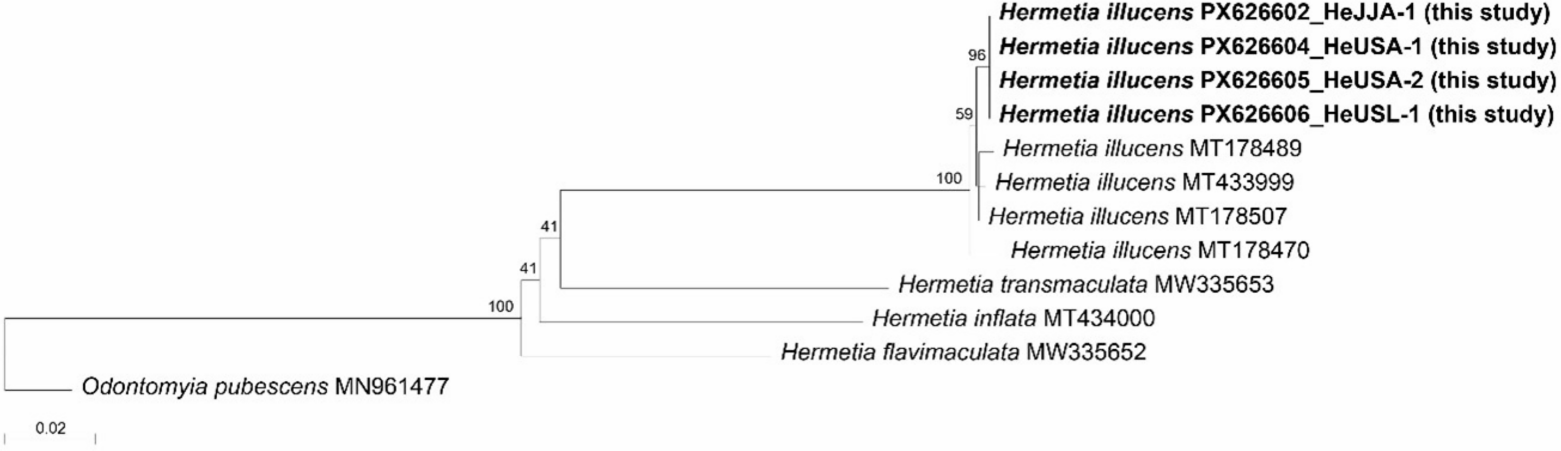
Phylogenetic tree constructed using the neighbor-joining (NJ) method showing the relationships among the 11 aligned *COI* sequences of *Hermetia* species. *Odontomyia pubescens* was selected as an outgroup. The numbers above the nodes represent NJ bootstrap values, expressed as percentages (%). Species names are indicated with the sequences’ accession numbers in the NCBI GenBank database, and the newly sequenced individual (in bold) is also labeled with its voucher number (see Table 3 for detailed information).

*Hermetia* larvae collected beneath a nest of captive *V. mandarinia* (HeUSL-1) (Fig. 6A), as well as adults that subsequently emerged from these larval aggregations (HeUSA-1, 2) (Fig. 6B), were identified using both morphological characteristics and COI barcode analyses (Fig. 7). All examined specimens were conclusively identified as *H. illucens*.

All larvae were found exclusively beneath the nests, where they were primarily observed feeding on fallen hornet carcasses and other decomposing organic material (Fig. 6C). No larvae were detected inside the nests.

### Genus *Volucella* (Diptera: Syrphidae)

**Figure 8:**
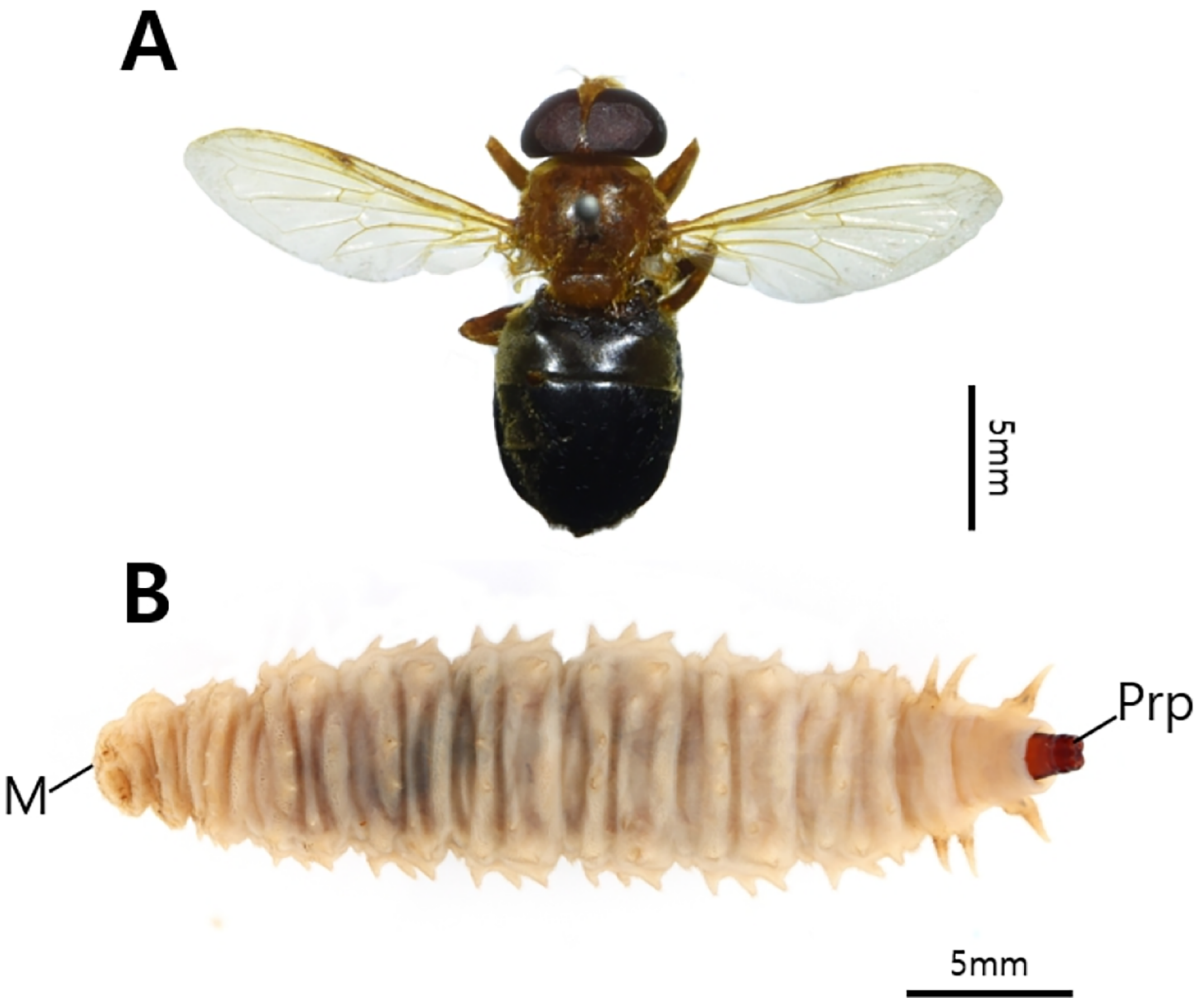
*Volucella suzukii*. (A) Adult (V-5) and (B) Larva (V-4), (M, mouth; Prp, posterior respiratory processes).

**Figure 9:**
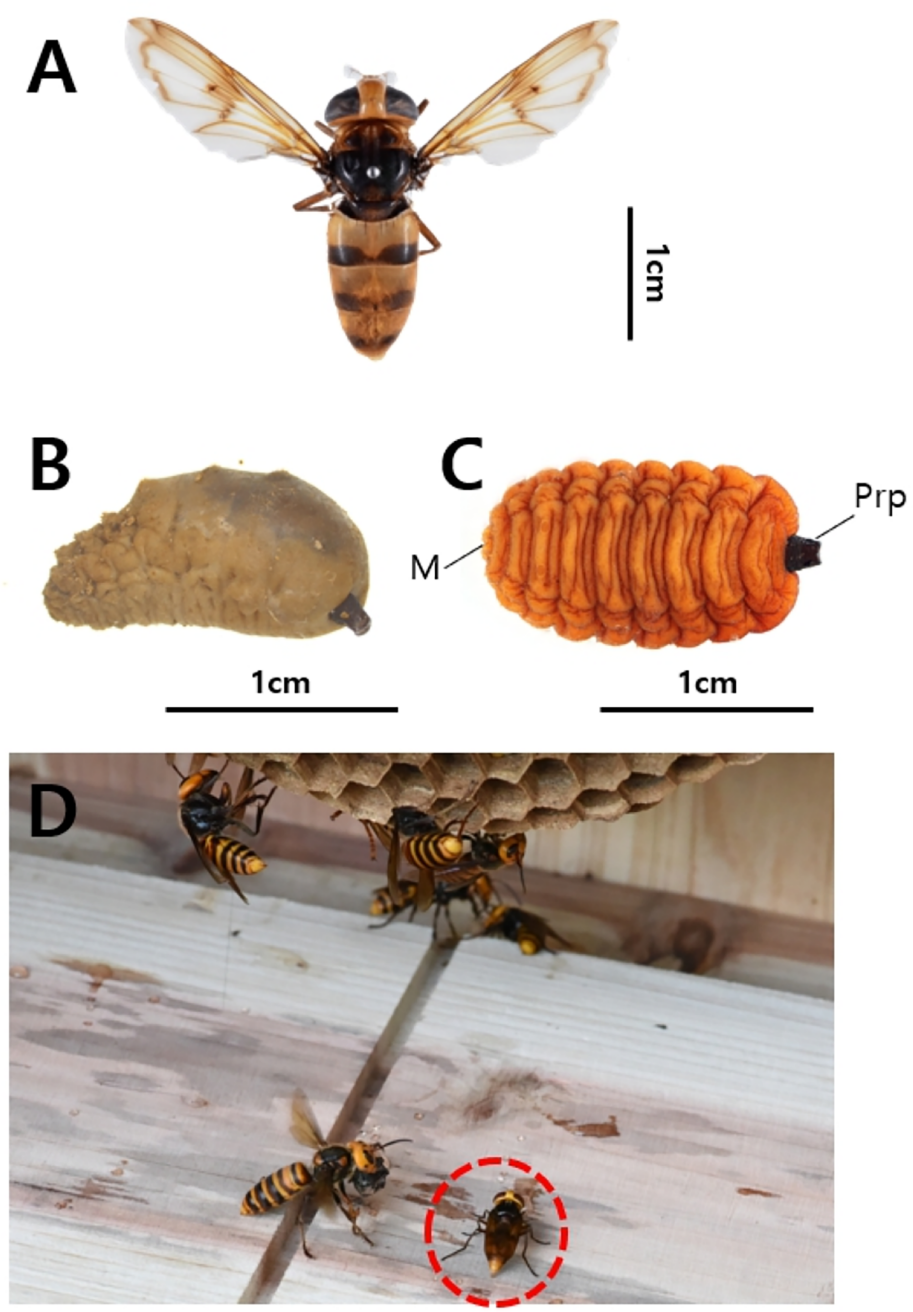
*Volucella coreana*. (A) Adult (V-15), (B) Pupa (V-12), and (C) Larva (V-6). (D) Photograph showing a *Vo. coreana* individual invading a reared *V*. *mandarinia* nest, (M, mouth; Prp, posterior respiratory processes).

*Volucella* spp. were collected from seven nests. Adult specimens were collected from Nest 2 (one individual, V-5) and Nest 7 (two individuals, V-15). Morphological analysis identified V-5 as *Vo. suzukii* Matsumura, 1916 (Fig. 8A), and V-15 as *Vo. coreana* Shiraki, 1930 (Fig. 9A).

**Figure 10:**
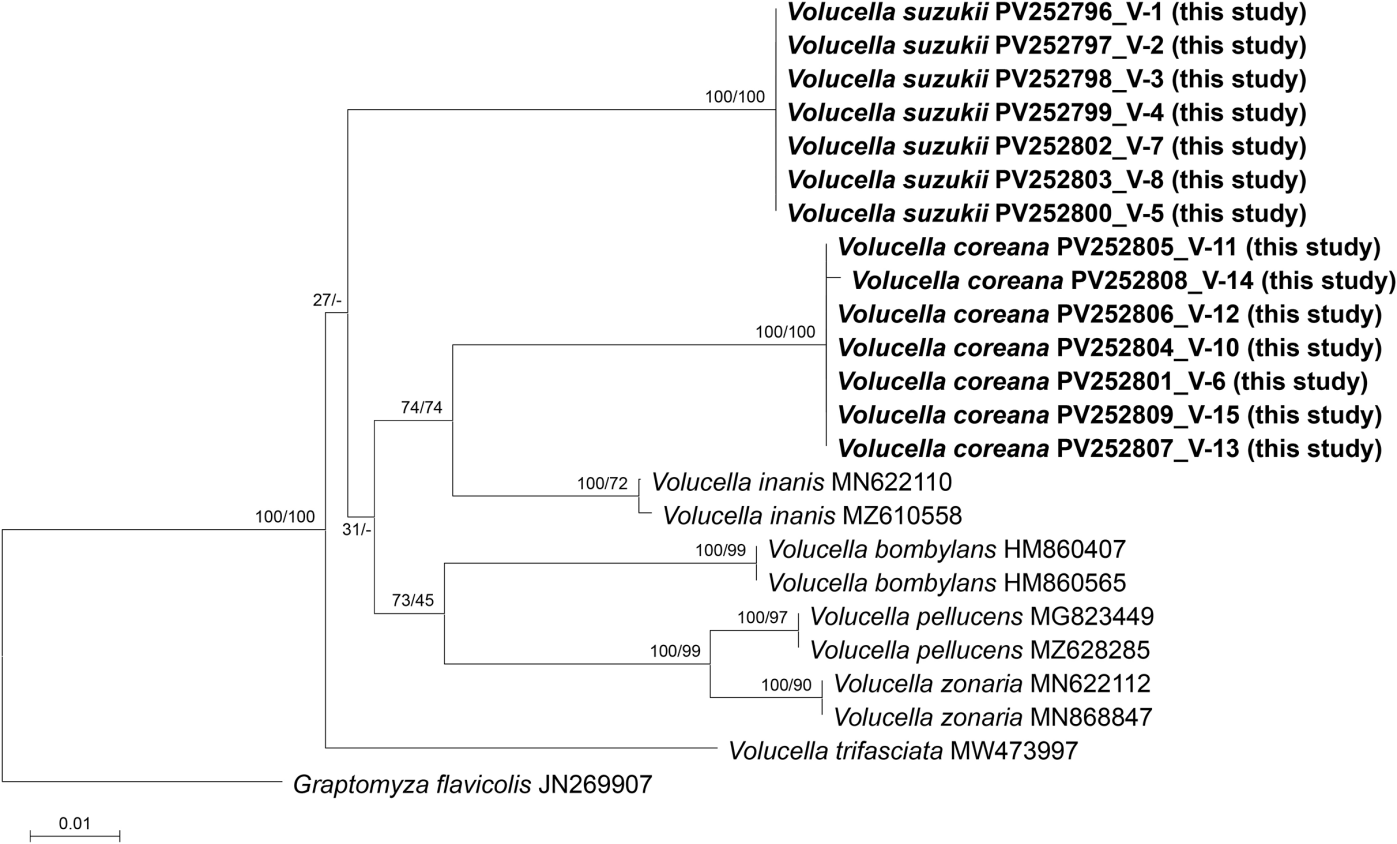
Phylogenetic tree constructed using the neighbor-joining (NJ) method showing the relationships among the 23 aligned *COI* sequences of *Volucella* species. *Graptomyza flavicolis* was selected as an outgroup. The numbers above the nodes represent NJ bootstrap values, expressed as percentages (%). Species names are indicated with the sequences’ accession numbers in the NCBI GenBank database, and the newly sequenced individual (in bold) is also labeled with its voucher number (see Table 3 for detailed information).

DNA barcoding of pupae and larvae identified larvae V-1, V-2, V-3, V-4, V-7, and V-8 as *Vo. suzukii* (Fig. 8B), whereas larvae V-6, V-10, V-11, V-13, and V-14, along with pupating individual V-12, were identified as *Vo. coreana* (Figs. 9B, 9C, 10). Specimen V-9 from Nest 4 could not be analyzed because of poor sample quality (Fig. 10).

The two species exhibited distinct spatial distributions within the nests. All *Vo. coreana* larvae were collected from cells within the comb, whereas *Vo. suzukii* larvae were found clustered on the ground beneath the nest. Both species were found cohabiting in Nest 2, indicating that multiple *Volucella* species can occur within a single *V. mandarinia* nest.

### Genus *Pyralis* (Lepidoptera: Pyralidae)

**Figure 11:**
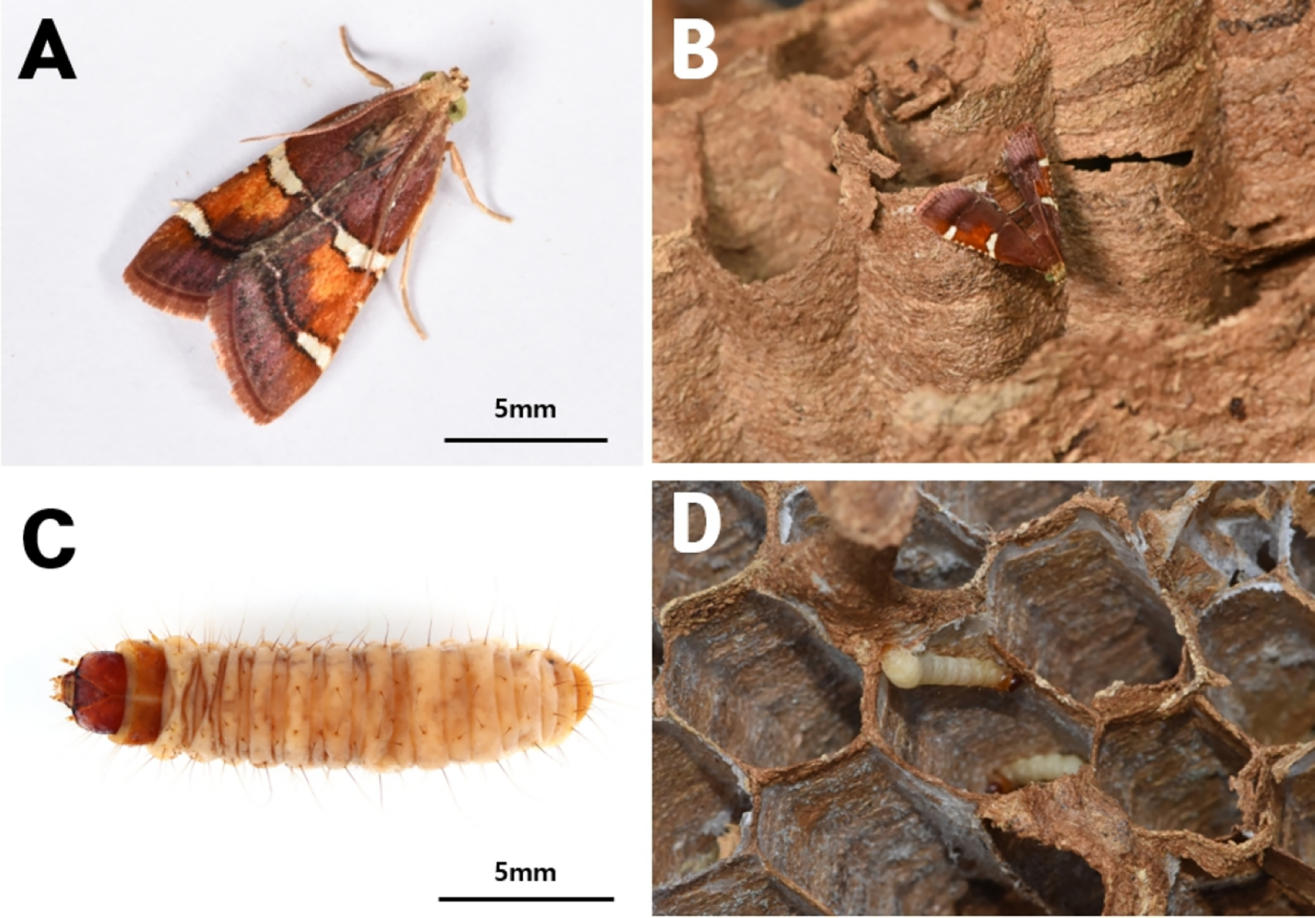
*Pyralis regalis*. (A) Adult, (B) an adult *P. regalis* moth after emerging from the nest, (C) larva, and (D) a larva inhabiting cells within the nest.

**Figure 12:**
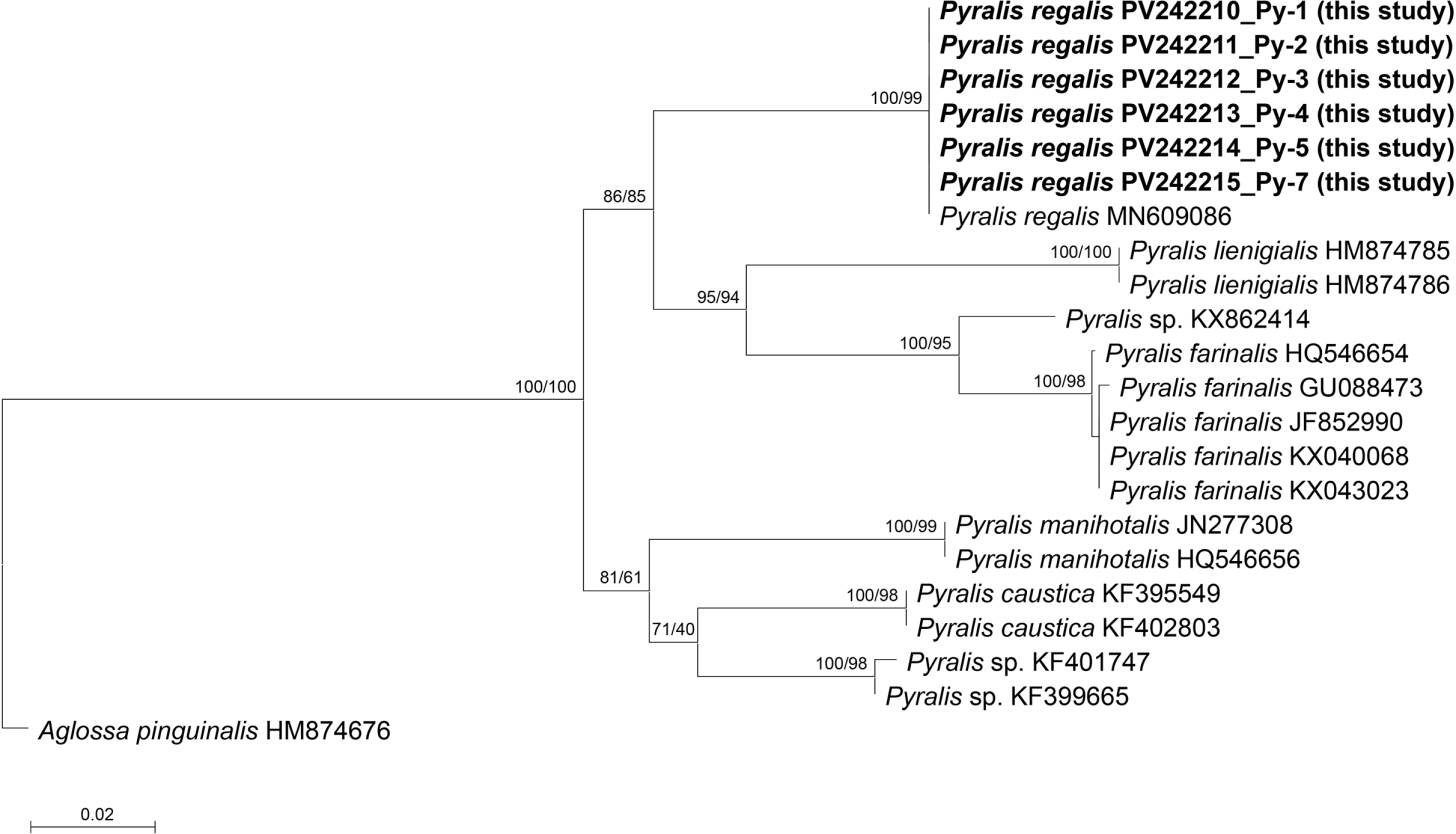
Phylogenetic tree constructed using the neighbor-joining (NJ) method showing the relationships among the 21 aligned *COI* sequences of *Pyralis* species. *Aglossa pinguinalis* was selected as an outgroup. The numbers above the nodes represent NJ bootstrap values, expressed as percentages (%). Species names are indicated with the sequences’ accession numbers in the NCBI GenBank database, and the newly sequenced individual (in bold) is also labeled with its voucher number (see Table 3 for detailed information).

Pyralid larvae (Fig. 11C) were collected from six nests. DNA barcoding of one larva from each nest confirmed that all six sequenced larvae were *Pyralis regalis* (Denis and Schiffermüller, 1775) (Fig. 12).

Additionally, adult pyralids that emerged in January and February 2022 from preserved nest samples (Nests 3 and 9) (Fig. 11A) were also identified as *P. regalis* based on morphological characteristics.

This species was recorded from six nests (Nests 3–5 and 7–9) collected in 2019 and 2021 from Ulsan, Gurye, and Andong, all initially obtained as larvae (Table 1; larval sequences Py-1 to Py-5 and Py-7). Two adult specimens subsequently emerged from larvae maintained in stored nest material in the laboratory during January and February 2022, with 38 and 45 individuals represented by vouchers Py-6 and Py-8, respectively (Table 1).

Larvae were primarily observed in the uppermost comb of *V. mandarinia* nests and later occupied older cells as development progressed. Individuals were observed burrowing within cells or moving along the nest surface (Fig. 11D). After pupation, adults remained inside the nest for a short period before exiting (Fig. 11B).

### Genus *Pheromermis* (Mermithida: Mermithidae)

**Figure 13:**
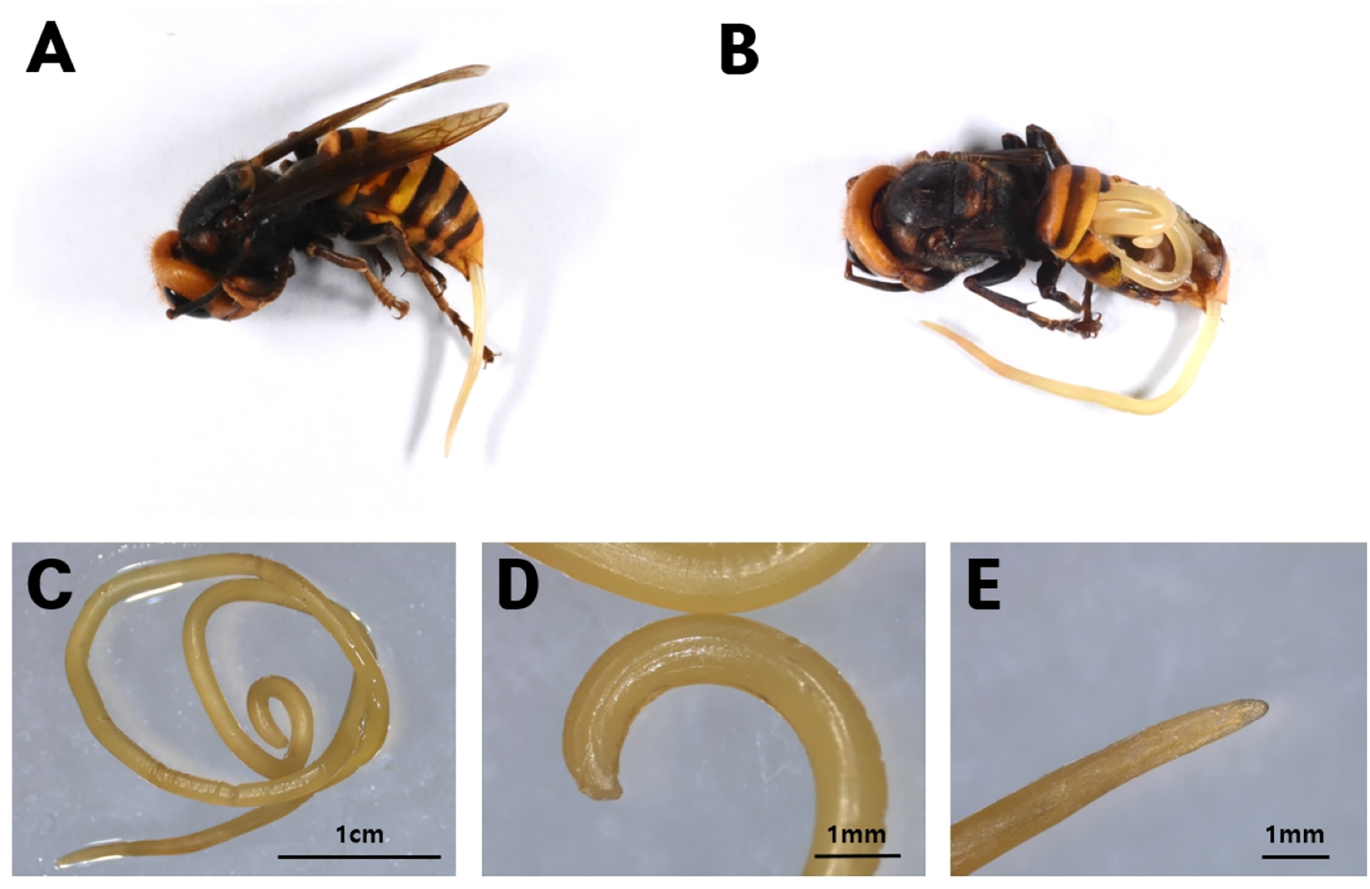
*Pheromermis vesparum*. (A) Protruding from the abdomen of a *V*. *mandarinia* queen. (B) The parasite in (A) observed through abdominal dissection. High magnification views of *Ph*. *vesparum*: (C) The whole worm, (D) tail, and (E) head.

A postparasitic juvenile nematode was observed partially emerging (approximately 10 mm) from the posterior end of the abdomen of a *V. mandarinia* queen (Fig. 13A). Dissection revealed that the nematode occupied most of the host’s abdominal cavity (Fig. 13B).

The nematode measured approximately 82.3 mm in length and exhibited a pale ivory coloration (Figs. 13C–13E). Based on DNA barcoding, the specimen was identified as *Ph. vesparum* Kaiser, 1987 (Mermithidae).

**Figure 14:**
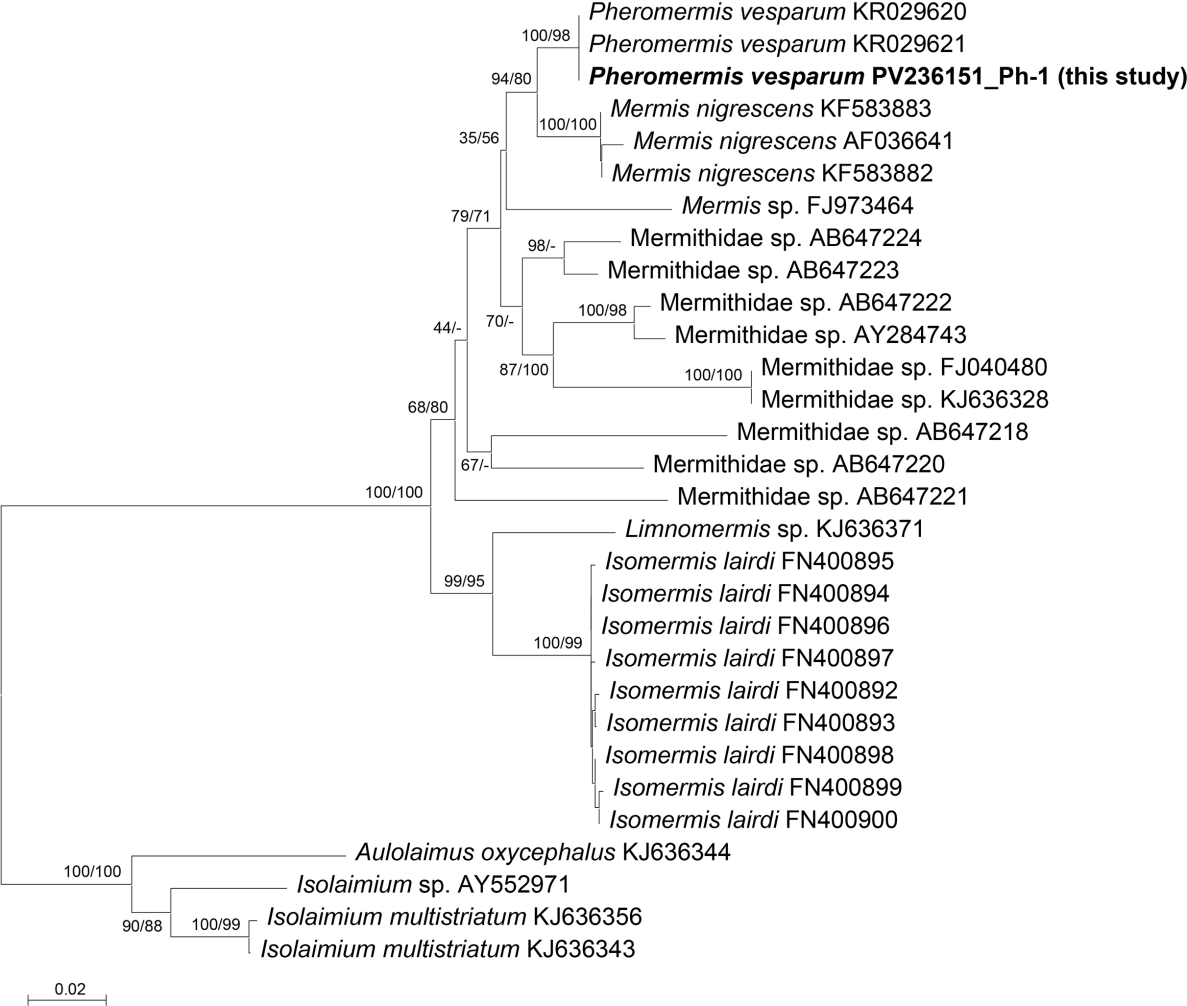
Phylogenetic tree constructed using the neighbor-joining (NJ) method showing the relationship among the 26 aligned *18S rRNA* sequences of mermithid species. Three species, *Aulolaimus oxycephalus*, *Isolaimium multistriatum*, and *Isolaimium* sp., were selected as outgroups. The numbers above the nodes represent NJ bootstrap values, expressed as percentages (%). Species names are indicated with the sequences’ accession numbers in the NCBI GenBank database, and the newly sequenced individual (in bold) is also labeled with its voucher number (see Table 3 for detailed information).

This represents the first record of the genus *Pheromermis* and the species *Ph. vesparum* in Korea (Fig. 14).

## Discussion

### Genus *Quedius* (Coleoptera: Staphylinidae)

Species of the genus *Quedius* (Staphylinidae) are frequently associated with nests of social wasps, typically inhabiting detritus-rich substrates beneath nests and feeding on organic debris, including fallen wasp remains (Nadolski, 2013; Ruchin et al., 2022). In Eurasia, *Q. dilatatus* and *Q. brevicornis* are frequently recorded in association with *V. crabro* nests. Larvae of *Q. brevicornis* primarily consume detritus but may occasionally prey on wasp larvae or pupae in the lower comb layers (Nadolski, 2013). In contrast, *Q. dilatatus* feeds exclusively on detritus and has not been shown to directly exploit *V. crabro* brood (Ruchin et al., 2022).

In Korea, both *Q. dilatatus* and *Q. pectinatus* have been reported (Cho, 1996); however, only *Q. pectinatus* was detected in the present study. *Q. pectinatus* was repeatedly found beneath *V. mandarinia* nests, including a dense population beneath a reared experimental nest. These observations suggest that this species commonly inhabits the detritus layer beneath hornet nests.

Neither larvae nor the highly active adults (Fig. 2B) were observed entering the nest structure or climbing onto the combs to prey on living hornets. These observations indicate that *Q. pectinatus* exploits nest-derived organic debris without directly interacting with or harming the host colony. Therefore, this species is best interpreted as a detritivore forming part of the symbiotic assemblage associated with *V. mandarinia* nests, rather than functioning as a predator or parasite of the hornet colony.

Although *Q. dilatatus* has been reported to prey on fly larvae within detritus beneath wasp nests (Ruchin et al., 2022), no evidence of competitive interactions or predator–prey relationships between *Q. pectinatus* and *Vo. suzukii* larvae was observed in the present study. Further investigations incorporating a larger number of natural nests and experimental approaches are required to clarify potential interspecific interactions among detritus-associated organisms within the symbiotic community of hornet nests.

Although *Q. dilatatus* was not detected in this study, its occurrence beneath hornet nests in Korea cannot be excluded. Surveys across a broader range of nesting environments may reveal its presence in similar ecological niches.

### Genus *Xenos* (Strepsipteran: Xenidae)

Nakase & Kato (2013) and Kim et al. (2025) recorded two strepsipteran species, *X. moutoni* and *X. oxyodontes*, parasitizing *Vespa* species in Korea. In the present study, only *X. moutoni* was identified from *V. mandarinia* (Fig. 5A). However, *X. moutoni* is the most frequently encountered strepsipteran species in Korea, and its morphological and ecological similarity to *X. oxyodontes* suggests that the latter may also be detected in future surveys of *Vespa* nests.

Strepsipterans exhibit haemocoelous viviparity, in which planidium larvae develop within the female’s abdomen by consuming maternal nutrients. Each female of *X. moutoni* produces approximately 30,000–35,000 larvae and releases approximately 1,000–2,000 larvae per day through the brood canal located on the cephalothorax. These larvae attach to *Vespa* foundresses or workers visiting sap sources and are subsequently transported to the nest, where they penetrate the host and initiate parasitism (Matsuura & Yamane, 1990).

Detailed accounts of the behavioral and physiological effects of stylopization have been documented primarily in *Xenos vesparum*–host systems (Beani et al., 2021), and comparable effects in the *X. moutoni*–*V. mandarinia* association remain poorly documented.

Based on studies of *X. vesparum* and related systems, stylopized hornets are known to cease labor activities such as nest construction, foraging, and brood care, and instead sustain themselves by feeding on tree sap (Makino, Yamaura & Yamauchi, 2010). In males and newly emerged queens, reproductive capacity is lost (Beani et al., 2021), leading to male mortality before winter, whereas stylopized queens and workers typically enter hibernation (Tatsuta & Makino, 2003). Notably, stylopized workers have been reported to exhibit extended lifespans, reappearing in spring alongside overwintered queens (Beani et al., 2021).

In this study, *X. moutoni*–infected hornets collected on 8 May (3.1 cm in body length; Fig. 3A①) and 15 June (2.8 cm; Fig. 3A②) were identified as workers. Given that *V. mandarinia* queens typically measure 3.8–4.3 cm in length (Looney et al., 2023), these individuals were most likely overwintered stylopized workers. Another host individual (X-3), measuring 4.0 cm in body length (Fig. 3A③), was identified as a stylopized queen. Because overwintered stylopized hornets remain independent during spring and eventually perish without contributing to nest establishment, their direct impact on colony founding is likely minimal.

Although strepsipterans do not directly cause immediate host mortality, high parasitism rates have been shown to reduce the effective workforce of hornet colonies, potentially resulting in smaller nest sizes (Makino, Yamaura & Yamauchi, 2010). Among parasites of social hornets, *Xenos* species are the most widespread and often exhibit relatively high infection rates. Nevertheless, given their generally low prevalence and sporadic occurrence at the population level, *Xenos* species are unlikely to function as effective biological control agents against *V. mandarinia*.

Although strepsipterans are known to infest all hornet castes (Makino & Yamashita, 1998), all *V. mandarinia* individuals parasitized by *X. moutoni* in this study were females, comprising one queen and seven workers (Table 1). No parasitized males were detected. This caste-biased pattern is consistent with the transmission ecology of strepsipteran parasites. Planidium larvae are transmitted to hosts at sap-feeding sites and other foraging locations, where workers and queens are far more active than males (Matsuura & Yamane, 1990; Makino & Yamashita, 1998). Male *V. mandarinia* rarely leave the nest except during mating flights and therefore have limited opportunities to encounter parasite larvae in the external environment. Although the ages of parasitized individuals could not be determined from the specimens examined, the observed caste-biased infection pattern suggests that external foraging activity is a key determinant of parasitism risk in *V. mandarinia*.

### Genus *Hermetia* (Diptera: Stratiomyidae)

In the present study, larvae of *H. illucens* were recorded exclusively beneath a reared *V. mandarinia* nest. Their spatial distribution and feeding behavior resembled those of *Vo. suzukii* and *Q. pectinatus*, both of which occupy detritus-rich substrates beneath hornet nests.

*H. illucens* is a well-known saprophagous species capable of efficiently exploiting a wide range of decomposing organic substrates, including food waste, and has recently attracted considerable attention as a model organism for organic waste recycling (Barragan-Fonseca, Dicke & van Loon, 2017). Given its strong adaptation to decaying organic matter, it is ecologically plausible that *Hermetia* larvae can persist beneath hornet nests, where organic debris and fallen insect remains commonly accumulate.

Larvae tentatively attributable to Stratiomyidae have previously been observed beneath natural *Vespa* nests in association with *Volucella* larval aggregations (M. B. Choi, unpublished data, observed on 23 August 2024). However, *H. illucens* has not yet been conclusively documented from natural *V. mandarinia* nests. Because the present record originates from a nest reared under artificial conditions, it remains uncertain whether *Hermetia* routinely colonizes hornet nests in natural environments. Nevertheless, given its ecological flexibility and saprophagous lifestyle, occasional colonization beneath hornet nests in natural settings cannot be excluded.

From an ecological perspective, *Hermetia* appears to function primarily as a scavenger within the detritus layer beneath hornet nests, exploiting nest-derived organic matter without directly interacting with the host colony through predation or other antagonistic behaviors. No antagonistic interactions with *Vo. suzukii* or *Q. pectinatus* larvae were observed, suggesting that these taxa may coexist within the same detritus-rich microhabitat as part of the symbiotic assemblage associated with *V. mandarinia* nests.

### Genus *Volucella* (Diptera: Syrphidae)

Ten species of *Volucella* have been recorded in Korea, and *Vo. suzukii* has previously been reported from nests of *V. mandarinia* (Kim & Kim, 1972). In the present study, *Vo. coreana* was newly identified from *V. mandarinia* nests (Fig. 9D), indicating that these two species are likely the *Volucella* taxa most frequently associated with *V. mandarinia* colonies in Korea.

**Figure 15:**
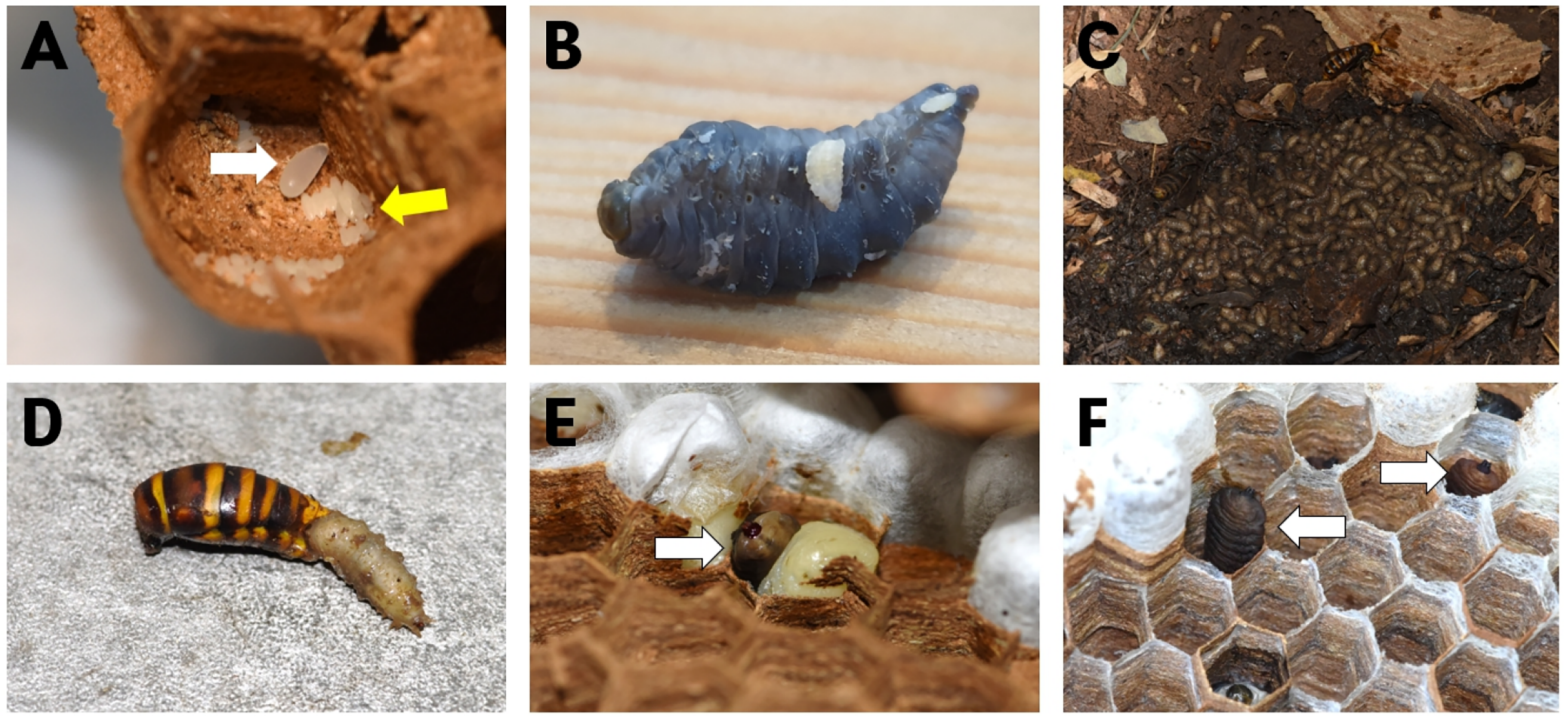
*Volucella suzukii* and *Vo*. *coreana* individuals inhabiting *Vespa mandarinia* nests. (A) *Volucella* eggs (yellow arrow) laid inside a nest cell alongside *V. mandarinia* eggs (white arrow), (B) early instar *Volucella* larvae feeding on a decomposing *V. mandarinia* larva, (C) *Vo. suzukii* larvae aggregated on the ground beneath a nest, (D) *Vo. suzukii* larvae feeding on the abdomen of a deceased *V. mandarinia* individual, (E) a *Vo. coreana* larva (white arrow) cohabiting with *V. mandarinia* larvae inside a nest cell, and (F) *Vo. coreana* larvae (white arrows) inhabiting multiple nest cells.

Several *Volucella* species are obligate sphecophiles that inhabit the nests of ground-nesting social wasps (Ball & Morris, 2015). Adult flies infiltrate nests to oviposit on the comb surface or within cells (Fig. 15A). In Europe, larvae of *Vo. zonaria* and *Vo. pellucens* typically descend beneath nests during development, where they scavenge detritus and wasp carcasses; they only occasionally remain within the nest and rarely prey on live wasp larvae (Rotheray & Gilbert, 1998). In the present study, *Vo. suzukii* exhibited comparable scavenging behavior beneath *V. mandarinia* nests (Figs. 15B–15D), and no predation on live hornet larvae or pupae was observed.

In contrast, *Vo. inanis* has been described in Europe as an obligate ectoparasite that resides primarily within the comb and actively preys on wasp larvae (Rupp, 1989; Brown, 2021a, 2021b). *Vo. coreana* showed a similar spatial association with the comb, including a flattened body form and persistent occupation of nest cells (Figs. 15E, 15F). Although direct predation was not observed during the limited observation periods of this study, its exclusive presence within the nest suggests the potential for facultative entomophagous behavior.

In New Zealand, *Vo. inanis* is currently being evaluated as a potential biological control agent against the invasive wasps *Vespula vulgaris* and *Vl. germanica* (Brown, 2021a, 2021b; Stratford et al., 2024). Despite their wide distribution in Korea, *Volucella* species remain poorly studied, with existing research largely restricted to adult taxonomy (Choi, 2004) and a morphological description of *Vo. suzukii* from Japan (Ohara, 1985). Further studies on larval development, feeding behavior, and host interactions are needed to clarify the ecological roles and potential impacts of Korean *Volucella* species within the symbiotic assemblage associated with *V. mandarinia* nests.

### Genus *Pyralis* (Lepidoptera: Pyralidae)

Shin et al. (2023) documented three moth species associated with social wasp nests in Korea, including *P. regalis*, which has been recorded from nests of 11 wasp species across three genera. Notably, *P. regalis* has been reported from all *Vespa* species in Korea except the rare *V. binghami* du Buysson.

Larvae of *Pyralis* species are generally regarded as facultative predators within wasp nests, primarily feeding on nest pulp and meconium but occasionally preying on wasp larvae or pupae (Miyano, 1980; Yamane, Fukuda & Makino, 2022). In polistine wasps with relatively small colonies, heavy infestations can lead to colony collapse because larval silk restricts adult wasp movement. In contrast, in larger *Vespa* colonies their overall impact is considered relatively minor (Shin et al., 2023).

In the present study, no direct predation on hornet larvae or pupae was observed, and *P. regalis* larvae were not attacked by adult hornets. These observations suggest that *P. regalis* exploits nest-derived organic material within *V. mandarinia* nests without causing substantial disruption to colony function.

From an ecological perspective, *P. regalis* appears to occupy an intermediate trophic role within the nest environment, functioning primarily as a detritus-associated organism while retaining the potential for facultative predatory behavior. Accordingly, this species may be regarded as part of the symbiotic assemblage associated with *V. mandarinia* nests, exploiting nest-derived substrates without exerting strong antagonistic effects on the host colony.

### Genus *Pheromermis* (Mermithida: Mermithidae)

*Ph. vesparum* has been reported primarily from Europe, with additional records from North America; there have been no confirmed records of this species from Asia (Kaiser, 1987; Molloy, Vinikour & Anderson, 1999). In Korea, mermithid nematodes are extremely rare, with only *Agamermis unka* Kaburaki & Jamamura documented to date (Choo, Kaya & Kim, 1995). Accordingly, the present study represents the first record of *Ph. vesparum* in Asia and the first record of the genus *Pheromermis* in the Korean fauna.

The specimen examined in this study was identified as a post-parasitic juvenile of *Ph. vesparum* based on DNA barcoding, comparison with reference sequences, and consistency with morphological descriptions provided by Villemant et al. (2015). This species is a parasitic nematode highly specialized for exploiting social wasps as definitive hosts (Kaiser, 1987).

Nematodes of the genus *Pheromermis* require a paratenic host to complete their life cycle. Adult nematodes oviposit in water or saturated soil, where larvae hatch and infect semi-aquatic insects, such as species belonging to Trichoptera, Plecoptera, Tipulidae, Ephemeroptera, and Coleoptera, which serve as paratenic hosts. Once these hosts mature, they may be captured by foraging worker wasps, processed into protein pellets, and fed to larvae within the nest, thereby enabling nematode transmission (Kaiser, 1987; Martin, 2004). Because wasps masticate prey into small fragments, nematode larvae within the digestive tracts of paratenic hosts are likely to survive ingestion and subsequently infect wasp larvae.

However, *V. mandarinia* predominantly preys on social bees and relatively large beetles, making the consumption of semi-aquatic insects, and thus exposure to *Pheromermis* larvae, unlikely (Matsuura & Yamane, 1990). This ecological trait may explain the extreme rarity of *Pheromermis* infections observed in Korean hornet populations.

Consistent with this interpretation, *Ph. vesparum* has been detected at very low prevalence in *V. velutina nigrithorax* Buysson populations in France (Villemant et al., 2015), indicating that *Pheromermis* infections are uncommon in social wasps. Although mermithid nematodes have occasionally been considered as potential biological control agents, reported infection rates are typically low, and even heavily parasitized colonies may persist and reproduce (Kaiser, 1987; Martin, 2004). Consequently, *Pheromermis* nematodes are unlikely to function as effective biological control agents against *Vespa* species at the population level.

### Functional classification of parasites, parasitoids, and nest-associated organisms

In this study, a total of seven species were identified inhabiting individuals or colonies of *V. mandarinia* in Korea. Based on their life-history traits and observed ecological interactions, *X. moutoni* and *Ph. vesparum* are best classified as obligate parasites that exert negative physiological effects on their hosts. Several authors have suggested that *Xenos* species may function as parasitoids of female Vespidae, as they induce reproductive death through castration and may indirectly contribute to host mortality (Kuris, 1974; Kathirithamby, 2009; Beani et al., 2017). However, this interpretation remains controversial (Eggleton & Belshaw, 1992), reflecting the complex and atypical physiological relationship between strepsipterans and their hosts.

In contrast, *Volucella* spp., *P. regalis*, *Q. pectinatus*, and *H. illucens* are more appropriately characterized as nest-associated organisms that exhibit varying degrees of niche partitioning within or beneath the hornet nest environment. *Vo. coreana* was consistently associated with the comb and nest cells, directly exploiting nest resources and potentially interacting with host brood; it may therefore be regarded as a facultative predator or parasitic inquiline. Conversely, *Vo. suzukii* remained beneath the nest throughout larval development, feeding on detritus and fallen organic material, and no direct predation on host individuals was observed. These observations are consistent with a commensal association, in which *Vo. suzukii* benefits from nest-derived resources without causing detectable harm to the host colony.

*H. illucens* exhibited an ecological role closely aligned with that of *Vo. suzukii*. Larvae were confined to detritus layers beneath reared nests and fed exclusively on decomposing organic matter, without entering the nest structure or attacking host individuals. Although its occurrence has only been confirmed under artificial rearing conditions, the observed behavior supports its interpretation as a scavenger-type, nest-associated organism. Assessing the potential benefit to the host colony through waste removal requires experimental verification.

*P. regalis* primarily feeds on nest pulp and meconium and has occasionally been reported to prey on wasp larvae or pupae. In the present study, however, no direct predation was observed, placing this species in an intermediate ecological position between a facultative predator and a nest-associated inquiline. Similarly, *Q. pectinatus* fed on organic debris beneath the nest and showed no evidence of direct host exploitation, supporting its classification as a commensal nest-associated species.

Despite their frequent presence within or beneath hornet nests, none of these four nest-associated species were observed to be actively attacked by adult *V. mandarinia*. Only circumstantial evidence suggests that *P. regalis* larvae may occasionally be consumed by hornet larvae (Shin et al., 2023). The apparent tolerance of these organisms by host workers suggests the possible involvement of chemical camouflage or mimicry, although this hypothesis remains to be tested experimentally.

### Geographic distribution patterns of parasites

In this study, the spatial distributions of *V. mandarinia* and its parasites showed limited overlap. *Vespa mandarinia* was collected throughout South Korea, whereas *Xenos* specimens were predominantly found in the eastern and northern regions, and a single *Ph. vesparum* specimen was collected from the southwestern region (Gurye, Jirisan area). Consequently, no geographic overlap between *Ph. vesparum* and *Xenos* parasitism was observed in the present study, precluding any assessment of potential combined or additive effects on host populations in areas where both parasites co-occur.

Although *V. mandarinia* is a native and highly abundant species in Korea, *Ph. vesparum* represents a new record for the Korean fauna and for Asia. Despite extensive surveys of social wasps conducted over several decades, involving the examination of thousands of hornet specimens, and despite comprehensive nationwide surveys by parasitologists, this nematode was detected for the first time in the present study. It remains unclear whether *Ph. vesparum* has recently colonized the Korean Peninsula or has long been present at extremely low prevalence but escaped detection. Additional records of parasitism are required to clarify the distribution, prevalence, and potential impact of these parasites on *V. mandarinia* populations in Korea. Furthermore, future studies examining geographic overlap between *V. mandarinia* and its parasites may provide valuable insights into the population-level effects of parasitism.

## Conclusions

This study documented the diversity and functional roles of parasites and nest-associated organisms associated with *V. mandarinia* colonies and individuals in Korea. Species were identified using morphological characteristics and DNA barcode analyses, and behavioral observations were used to infer their ecological roles within and around the nest environment.

The results indicate that the strepsipteran *X. moutoni* alters host behavior but is unlikely to function as an effective biological control agent at the population level. The nematode *Ph. vesparum*, recorded here for the first time in Asia and Korea, was detected in only a single individual, suggesting a negligible impact on *V. mandarinia* populations under natural conditions.

Among nest-associated insects, *Vo. suzukii*, *Q. pectinatus*, and *H. illucens* primarily exploited detritus and decomposing organic material beneath nests and showed no evidence of direct predation on host individuals, suggesting primarily commensal associations. *Vo. coreana* differed from these species in its close association with the comb and nest cells and may occasionally exploit host resources within the nest, including brood or other organic material, suggesting a more parasitic or predatory tendency. *P. regalis* occupied an intermediate ecological position, primarily feeding on nest material and meconium, with limited direct effects on colony maintenance observed in this study.

Overall, these findings highlight the ecological complexity of hornet nests as microhabitats that support a diverse assemblage of parasites and nest-associated organisms. Most associated species appear to have limited direct influence on host population regulation, instead occupying specialized niches within or beneath the nest environment. This study provides a baseline framework for future investigations into the ecological interactions, host specificity, and chemical integration mechanisms of nest-associated organisms, which will be essential for evaluating their potential direct or indirect roles in the population dynamics and management of *V. mandarinia*.

## Supplemental Information

10.7717/peerj.21240/supp-1 Supplemental Information 1 COI and 18S rRNA sequences generated in this study (FASTA) (Pheromermis vesparum). FASTA-formatted DNA barcode (COI) and 18S rRNA sequences generated in this study (*Pheromermis vesparum*).

10.7717/peerj.21240/supp-2 Supplemental Information 2 COI and 18S rRNA sequences of *Hermetia illucens* compiled in FASTA format, including additional sequences used for sequence comparison.

10.7717/peerj.21240/supp-3 Supplemental Information 3 COI and 18S rRNA sequences generated in this study (FASTA) (Pyralis spp). FASTA-formatted DNA barcode (COI) and 18S rRNA sequences generated in this study (*Pyralis* spp).

10.7717/peerj.21240/supp-4 Supplemental Information 4 Multiple sequence alignments used for phylogenetic analyses (FASTA) (Hermetia spp.). FASTA-formatted multiple sequence alignments (trimmed) used to build the phylogenetic trees reported (*Hermetia* spp.).

10.7717/peerj.21240/supp-5 Supplemental Information 5 COI and 18S rRNA sequences generated in this study (FASTA). FASTA-formatted DNA barcode (COI) and 18S rRNA sequences generated in this study.

10.7717/peerj.21240/supp-6 Supplemental Information 6 COI and 18S rRNA sequences generated in this study (FASTA) (Pyralis spp.). FASTA-formatted DNA barcode (COI) and 18S rRNA sequences generated in this study, provided as supplementary files for peer review (*Pyralis* spp.).

10.7717/peerj.21240/supp-7 Supplemental Information 7 Multiple sequence alignments used for phylogenetic analyses (FASTA) (Nematoda). FASTA-formatted multiple sequence alignments (trimmed) used to build the phylogenetic trees reported (Nematoda).

10.7717/peerj.21240/supp-8 Supplemental Information 8 COI and 18S rRNA sequences generated in this study (FASTA) (Volucella spp.). FASTA-formatted DNA barcode (COI) and 18S rRNA sequences generated in this study (*Volucella* spp.).

10.7717/peerj.21240/supp-9 Supplemental Information 9 COI and 18S rRNA sequences generated in this study (FASTA) (*Hermetia illucens*). FASTA-formatted DNA barcode (COI) and 18S rRNA sequences generated in this study (*Hermetia illucens*).

10.7717/peerj.21240/supp-10 Supplemental Information 10 COI and 18S rRNA sequences generated in this study (FASTA) (*Xenos moutoni*). FASTA-formatted DNA barcode (COI) and 18S rRNA sequences generated in this study (*Xenos moutoni*).

10.7717/peerj.21240/supp-11 Supplemental Information 11 COI and 18S rRNA sequences generated in this study (FASTA) (Quedius spp.). FASTA-formatted DNA barcode (COI) and 18S rRNA sequences generated in this study (*Quedius* spp.).

10.7717/peerj.21240/supp-12 Supplemental Information 12 Multiple sequence alignments used for phylogenetic analyses (FASTA) (Volucella spp.). FASTA-formatted multiple sequence alignments (trimmed) used to build the phylogenetic trees reported (*Volucella* spp.).

10.7717/peerj.21240/supp-13 Supplemental Information 13 Specimen-level collection records underlying Table 1 (raw data). Specimen-level collection records used in this study, including collection source (trapped individuals or nests), locality, collection date, host taxon, associated organisms identified, and related metadata. The raw data underlying Table 1 and associated analyses.

## Additional Information and Declarations

### DNA Deposition

The following information was supplied regarding the deposition of DNA sequences:

The DNA barcode (COI) and 18S rRNA sequences generated in this study are available at NCBI GenBank: PV246623–PV246631, PV252796–PV252809, PV242210–PV242215, PV241484–PV241487, PX626602, PX626604–PX626606, and PV236151.

### Data Availability

The following information was supplied regarding data availability:

The specimen-level collection records, including collection source (trapped individuals or nests), locality, date, and associated metadata, underlying Table 1 are available in the Supplemental File.

## References

[ref-1] Abe T, Kawai N, Niwa A (1982). Purification and properties of a presynaptically acting neurotoxin, mandaratoxin, from hornet (*Vespa mandarinia*). Biochemistry.

[ref-2] Alaniz AJ, Carvajal MA, Vergara PM (2021). Giants are coming? Predicting the potential spread and impacts of the giant Asian hornet (*Vespa mandarinia*, Hymenoptera: Vespidae) in the USA. Pest Management Science.

[ref-3] Archer ME (2012). Vespine wasps of the world: behaviour, ecology & taxonomy of the vespinae.

[ref-4] Ball S, Morris R (2015). Britain’s Hoverflies a field guide-revised and updated.

[ref-5] Barragan-Fonseca KB, Dicke M, van Loon JJ (2017). Nutritional value of the black soldier fly (Hermetia illucens L.) and its suitability as animal feed-a review. Journal of Insects as Food and Feed.

[ref-6] Beani L, Dallai R, Cappa F, Manfredini F, Zaccaroni M, Lorenzi MC, Mercati D (2021). A strepsipteran parasite extends the lifespan of workers in a social wasp. Scientific Reports.

[ref-7] Beani L, Marchini D, Cappa F, Petrocelli I, Gottardo M, Manfredini F, Giusti F, Dallai R (2017). Subtle effect of *Xenos vesparum* (Xenidae, Strepsiptera) on the reproductive apparatus of its male host: parasite or parasitoid?. Journal of Insect Physiology.

[ref-8] Brown RL (2021a). Approvals to release biocontrol agents, Landcare Research. https://www.landcareresearch.co.nz/discover-our-research/biodiversity-biosecurity/invasive-invertebrates/approvals-to-release-biocontrol-agents/.

[ref-9] Brown RL (2021b). *Volucella inanis* host test results, Landcare Research. https://www.landcareresearch.co.nz/assets/Discover-Our-Research/Biosecurity/Invasive-invertebrates/EPA-applications/Summary_host_testing_Volucella_inanis_results.pdf.

[ref-10] Cho YB (1996). Taxonomic study on the tribe Quediini (Coleoptera, Staphylinidae) from Korea. Korean Journal of Systematic Zoology.

[ref-11] Choi DS (2004). A systematic study of Korean *Volucella* (Diptera: Syrphidae) and ecological monitoring of butterflies. Ph.D dissertation. Yonsei University, Seoul, South Korea.

[ref-12] Choi MB, Kim TG, Kwon O (2019). Recent trends in wasp nest removal and hymenoptera stings in South Korea. Journal of Medical Entomology.

[ref-13] Choi MB, Kwon O (2015). Occurrence of Hymenoptera (wasps and bees) and their foraging in the southwestern part of Jirisan National Park, South Korea. Journal of Ecology and Environment.

[ref-14] Choi MB, Kim JK, Lee JW (2012). Increase trend of social hymenoptera (wasps and honeybees) in urban areas, inferred from moving-out case by 119 rescue services in Seoul of South Korea. Entomological Research.

[ref-15] Choo HY, Kaya H, Kim HH (1995). Biological studies on *Agamermis unka* (Nematoda: Mermithidae), a Parasite of the Brown Planthopper *Nilaparvata lugens*. Biocontrol Science and Technology.

[ref-16] Eggleton P, Belshaw R (1992). Insect parasitoids: an evolutionary overview. Philosophical Transactions of the Royal Society of London. Series B.

[ref-17] Folmer O, Black M, Hoeh W, Lutz R, Vrijenhoek R (1994). DNA primers for amplification of mitochondrial cytochrome c oxidase subunit I from diverse metazoan invertebrates. Molecular Marine Biology and Biotechnology.

[ref-18] Giribet G, Carranza S, Baguna J, Ruitort M, Ribera C (1996). First molecular evidence for the existence of a Tardigrada + Arthropoda clade. Molecular Biology and Evolution.

[ref-19] Hirano K, Tanikawa A (2020). Ocular injury caused by the sprayed venom of the Asian Giant Hornet (*Vespa mandarinia*). Case Reports in Ophthalmology.

[ref-20] Jeong G, Kang H, Choi H, Lee Y, Jin SD (2018). External morphology and habitat of black soldier fly (*Hermetia illucens* L.) in Korea. Korean Journal of Environmental Biology.

[ref-21] Jung C, Kang MS, Kim DW, Lee HS (2007). Vespid wasps (Hymenoptera) occurring around apiaries in Andong, Korea. Korea Journal of Apiculture.

[ref-22] Kaiser H (1987). Biologie, Okologie und Entwicklung des europaischen Wespen-Parasitoiden *Pheromermis vesparum* n. sp. (Mermithidae, Nematoda). Zoologische Jahrbucher. Abteilung fur Systematik, Okologie und Geographie der Tiere.

[ref-23] Kathirithamby J (2009). Host-parasitoid associations in Strepsiptera. Annual Review of Entomology.

[ref-24] Kim C, Choi MB (2021). Distribution of social wasps in two Metropolitan Cities (Busan and Daegu) of South Korea. PNIE.

[ref-25] Kim JI, Kim C-W (1972). On the 17 unrecorded Syrphidae (Dipt.) from Korea. Korean Journal of Entomology.

[ref-26] Kim I-K, Kim C-J, Choi J-H, Kang HJ, Choi MB (2025). Stylopization by *Xenos* spp. (Xenidae, Strepsiptera) in invasive alien hornet, *Vespa velutina*, in South Korea. Parasite.

[ref-27] Kim WM, Kim SY, Song W (2020). Microhabitat characteristics affecting the occurrence and diversity of queen hornets (genus *Vespa*) in an urban green area. Landscape and Ecological Engineering.

[ref-28] Kumar S, Stecher G, Suleski M, Sanderford M, Sharma S, Tamura K (2024). Molecular evolutionary genetics analysis version 12 for adaptive and green computing. Molecular Biology and Evolution.

[ref-29] Kuris AM (1974). Trophic interactions: similarity of parasitic castrators to parasitoids. The Quarterly Review of Biology.

[ref-30] Kwon O, Choi MB (2020). Interspecific hierarchies from aggressiveness and body size among the invasive alien hornet, *Vespa velutina nigrithorax*, and five native hornets in South Korea. PLOS ONE.

[ref-31] Looney C, Carman B, Cena J, Cichorz C, Iyer V, Orr J, Roueché N, Salp K, Serrano JM, Udo L, van Westendorp P, Wilson TM, Wojahn R, Spichiger S-E (2023). Detection and description of four *Vespa mandarinia* (Hymenoptera, Vespidae) nests in western North America. Journal of Hymenoptera Research.

[ref-32] Makino S, Kawashima M, Kosaka H (2011). First record of occurrence of *Xenos moutoni* (Strepsiptera: Stylopidae), an important parasite of hornets (Hymenoptera: Vespidae: *Vespa*), in Korea. Journal of Asia-Pacific Entomology.

[ref-33] Makino S, Yamashita Y (1998). Levels of parasitism by *Xenos moutoni* du Buysson (Strepsiptera, Stylopidae) and their seasonal changes in hornets (Hymenoptera: Vespidae, Vespa) caught with bait traps. Entomological Science.

[ref-34] Makino S, Yamaura Y, Yamauchi H (2010). Smaller nests of the hornet *Vespa analis* (Hymenoptera, Vespidae) are more severely affected by the strepsipteran parasite *Xenos moutoni* (Strepsiptera, Stylopidae) than are larger nests. Insectes Sociaux.

[ref-35] Martin SJ (2004). A simulation model of biological control of social wasps (Vespinae) using mermithid nematodes. New Zealand Journal of Zoology.

[ref-36] Matsuura M (1984). Comparative biology of the five Japanese species of the genus *Vespa* (Hymenoptera, Vespidae). Bulletin of the Faculty of Agriculture, Mie University.

[ref-37] Matsuura M, Sakagami S (1973). A bionomic sketch of the Giant Hornet, *Vespa mandarinia*, a serious pest for Japanese Apiculture. Journal of the Faculty of Science, Hokkaido University Series.

[ref-38] Matsuura M, Yamane S (1990). Biology of the vespine wasps.

[ref-39] Miyano S (1980). Life tables of colonies and workers in a paper wasp, *Polistes chinensis antennalis*, in central Japan (Hymenoptera: Vespidae). Researches on Population Ecology.

[ref-40] Molloy DP, Vinikour WS, Anderson RV (1999). New North American records of aquatic insects as paratenic hosts of *Pheromermis* (Nematoda: Mermithidae). Journal of Invertebrate Pathology.

[ref-41] Nadolski J (2013). Factors restricting the abundance of wasp colonies of the European hornet Vespa crabro and the Saxon wasp *Dolichovespula saxonica* (Hymenoptera: Vespidae) in an urban area in Poland. Entomologica Fennica.

[ref-42] Nakase Y, Kato M (2013). Cryptic diversity and host specificity in giant *Xenos* strepsipterans parasitic in large *Vespa* hornets. Zoological Science.

[ref-43] Nuñez-Penichet C, Osorio-Olvera L, Gonzalez VH, Cobos ME, Jiménez L, DeRaad DA, Alkishe A, Contreras-Díaz RG, Nava-Bolaños A, Utsumi K, Ashraf U, Adeboje A, Peterson AT, Soberon J (2021). Geographic potential of the world’s largest hornet, *Vespa mandarinia* Smith (Hymenoptera: Vespidae), worldwide and particularly in North America. PeerJ.

[ref-44] Ohara K (1985). The larvae and puparia of four Japanese species of the genus *Volucella* Geoffroy (Diptera: Syrphidae), II. Kontyu.

[ref-45] Ono M, Igarashi T, Ohno E, Sasaki M (1995). Unusual thermal defence by a honeybee against mass attack by hornets. Nature.

[ref-46] Rotheray G, Gilbert F (1998). Phylogeny of Palaearctic Syrphidae (Diptera): evidence from larval stages. Zoological Journal of the Linnean Society.

[ref-47] Ruchin AB, Egorov LV, Solodovnikov AY, Antropov AV (2022). Abundance patterns of *Quedius dilatatus* leach (Coleoptera, Staphylinidae) and *Vespa crabro* L. (Hymenoptera, Vespidae) in Central European Russia suggest close adaptation of the Inquiline Rove Beetle life cycle to the nest dynamics of its wasp host. Entomological Review.

[ref-48] Rupp L (1989). The central European species of the genus Volucella (Diptera, Syrphidae) as commensals and parasitoids in the nests of bees and social wasps: studies on host-finding, larval biology and mimicry.

[ref-49] Shin Y-M, Lee HS, Kim IK, Kim C-J, Choi MB (2023). Host range expansion of nest-parasitic moths *Pyralis regalis* and *Hypsopygia mauritialis* in social wasp nests: new findings and implications for biological control. Diversity.

[ref-50] Smith-Pardo AH, Carpenter JM, Kimsey L (2020). The diversity of hornets in the genus *Vespa* (Hymenoptera: Vespidae; Vespinae), their importance and interceptions in the United States. Insect Systematics and Diversity.

[ref-51] Stratford JE, Stratford FMW, Brown RL, Oi CA (2024). Nest visitors of *Vespula* wasps and their potential use for biological control in an invaded range. Journal of Pest Science.

[ref-52] Tatsuta H, Makino S (2003). Rate of strepsipteran parasitization among overwintered females of the hornet *Vespa analis* (Hymenoptera: Vespidae). Environmental Entomology.

[ref-53] Villemant C, Zuccon D, Rome Q, Muller F, Poinar GO, Justine JL (2015). Can parasites halt the invader? Mermithid nematodes parasitizing the yellow-legged Asian hornet in France. PeerJ.

[ref-54] Watanabe Y, Arai K (2015). A new species of *Quedius* (Coleoptera, Staphylinidae) Obtained from a Nest of *Vespa mandarinia* in Kobe, Japan. Elytra, Tokyo, New Series.

[ref-55] Wilson TM, Takahashi J, Spichiger S-E, Kim I, van Westendorp P (2020). First reports of *Vespa mandarinia* (Hymenoptera: Vespidae) in North America represent two separate maternal lineages in Washington State, United States, and British Columbia, Canada. Annals of the Entomological Society of America.

[ref-56] Yamane S, Fukuda T, Makino S (2022). Observations on a nest of a paper wasp (*Polistes* sp.) infested by a pyralid moth, *Hypsopygia mauritialis* (Boisduval, 1833). Lepidoptera Science.

[ref-57] Yanagawa Y, Morita K, Sugiura T, Okada Y (2007). Cutaneous hemorrhage or necrosis findings after *Vespa mandarinia* (wasp) stings may predict the occurrence of multiple organ injury: a case report and review of literature. Clinical Toxicology.

[ref-58] Zhao Z-Y, Zhou H-Z (2015). Phylogeny and taxonomic revision of the subgenus *Velleius* Leach (Coleoptera: Staphylinidae: Staphylininae). Zootaxa.

